# Structure-Activity Relationships of Novel Salicylaldehyde Isonicotinoyl Hydrazone (SIH) Analogs: Iron Chelation, Anti-Oxidant and Cytotoxic Properties

**DOI:** 10.1371/journal.pone.0112059

**Published:** 2014-11-13

**Authors:** Eliška Potůčková, Kateřina Hrušková, Jan Bureš, Petra Kovaříková, Iva A. Špirková, Kateřina Pravdíková, Lucie Kolbabová, Tereza Hergeselová, Pavlína Hašková, Hana Jansová, Miloslav Macháček, Anna Jirkovská, Vera Richardson, Darius J. R. Lane, Danuta S. Kalinowski, Des R. Richardson, Kateřina Vávrová, Tomáš Šimůnek

**Affiliations:** 1 Charles University in Prague, Faculty of Pharmacy in Hradec Králové, Hradec Králové, Czech Republic; 2 Molecular Pharmacology and Pathology Program, Bosch Institute and Department of Pathology, University of Sydney, Sydney, Australia; Lady Davis Institute for Medical Research/McGill University, Canada

## Abstract

Salicylaldehyde isonicotinoyl hydrazone (SIH) is a lipophilic, tridentate iron chelator with marked anti-oxidant and modest cytotoxic activity against neoplastic cells. However, it has poor stability in an aqueous environment due to the rapid hydrolysis of its hydrazone bond. In this study, we synthesized a series of new SIH analogs (based on previously described aromatic ketones with improved hydrolytic stability). Their structure-activity relationships were assessed with respect to their stability in plasma, iron chelation efficacy, redox effects and cytotoxic activity against MCF-7 breast adenocarcinoma cells. Furthermore, studies assessed the cytotoxicity of these chelators and their ability to afford protection against hydrogen peroxide-induced oxidative injury in H9c2 cardiomyoblasts. The ligands with a reduced hydrazone bond, or the presence of bulky alkyl substituents near the hydrazone bond, showed severely limited biological activity. The introduction of a bromine substituent increased ligand-induced cytotoxicity to both cancer cells and H9c2 cardiomyoblasts. A similar effect was observed when the phenolic ring was exchanged with pyridine (*i.e.*, changing the ligating site from *O*, *N*, *O* to *N*, *N*, *O*), which led to pro-oxidative effects. In contrast, compounds with long, flexible alkyl chains adjacent to the hydrazone bond exhibited specific cytotoxic effects against MCF-7 breast adenocarcinoma cells and low toxicity against H9c2 cardiomyoblasts. Hence, this study highlights important structure-activity relationships and provides insight into the further development of aroylhydrazone iron chelators with more potent and selective anti-neoplastic effects.

## Introduction

Iron is a crucial component of various proteins involved in oxygen transport, cellular respiration, metabolism and division [1], [2], [3]. The majority of cellular iron acquired by tumor cells is stored in ferritin [4], [5], with smaller amounts being utilized for cellular metabolism, such as the synthesis of heme or iron-sulfur clusters [6], [7]. Intracellular iron is also found within a poorly defined “labile iron pool” (LIP), in which iron may be in transit between proteins and/or low-molecular weight (*M*_r_) ligands, or specifically transported by putative iron-chaperone proteins, such as poly(rC)-binding proteins 1–4 [8], [9].

When intracellular iron is depleted, the synthesis of new iron-dependent proteins and enzymes, and the processes they regulate (*e.g.*, cellular growth and proliferation), can be inhibited [10], [11]. On the other hand, when iron is present in excess, iron-mediated oxidative stress can lead to the damage of proteins, lipids and nucleic acids and can be cytotoxic. In fact, “free” or labile redox-active iron can catalyze the Fenton and Haber-Weiss-type reactions that generate highly toxic reactive oxygen species (ROS) [2], [4]. Classical iron chelators used in the clinics, such as desferrioxamine (DFO), deferiprone, and deferasirox, sequester iron and are primarily used to manage disorders with increased systemic iron levels, such as that caused by repeated blood transfusions in β-thalassemia major patients [12], [13], [14]. More recently, iron chelators have been also studied in pathological conditions associated with oxidative stress unrelated to iron-overload diseases [15].

Cancer cells require more iron than their neoplastic counterparts in order to support their increased rates of proliferation [1]. Indeed, iron is a key cofactor of ribonucleotide reductase, an enzyme that catalyzes the rate-limiting step in DNA synthesis [16], [17]. Cancer cells up-regulate transferrin (Tf) receptor 1 (TfR1) expression on their surface to increase iron uptake from the iron transport protein, Tf [18], [19]. Some cancer cells also express hepcidin, a hormone that induces the internalization of the iron-export protein, ferroportin 1, leading to reduced iron efflux from cells [19], [20]. Iron chelators induce iron depletion with subsequent G_1_-S cell cycle arrest and apoptosis [21] and they are increasingly studied as potential anti-neoplastic agents, with several in pre-clinical or clinical development [13], [22], [23].

**Figure 1:**
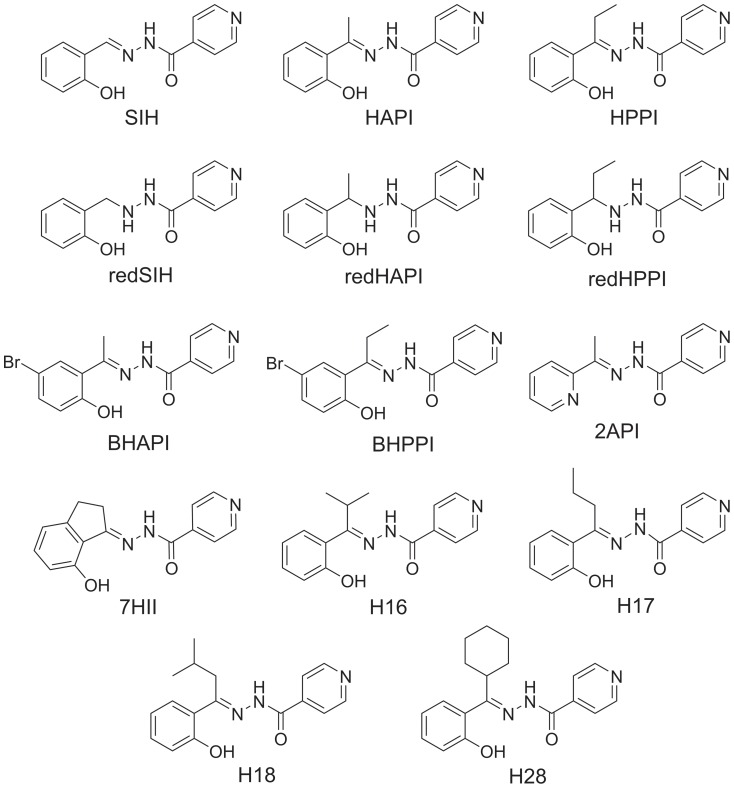
Line drawings of the chemical structures of the iron chelators, SIH, HAPI and HPPI, and their novel analogs.

*N′-*Salicylaldehyde isonicotinoyl hydrazone (SIH, Fig. 1) is a well-established tridentate iron chelator, which forms 2∶1 complexes with both Fe^3+^ and Fe^2+^ ions [24], [25]. SIH has been shown to: (***1***) protect various cell types against oxidative stress-inducing agents [15], [26], [27]; (***2***) prevent the cardiotoxicity of anthracycline-based antineoplastic agents both *in vitro* and *in vivo* [28]; and (***3***) act as a potential radio-protective, anti-viral and anti-cancer agent [29], [30], [31]. SIH has low *in vitro* and *in vivo* toxicity and good tolerability, even following prolonged administration to animals [32]. Recently, a series of new analogs of SIH were developed that have markedly enhanced hydrolytic stability compared to SIH and retain their ability to protect cells against oxidative injury [33]. In addition, these agents have increased cytotoxic activity compared to SIH [31]. The lead ligands identified in this series included (*E*)-*N′*-[1-(2-hydroxyphenyl)ethylidene]isonicotinoylhydrazide (HAPI; Fig. 1) and (*E*)-*N′*-[1-(2-hydroxyphenyl)propylidene]isonicotinoylhydrazide (HPPI; Fig. 1), which possess either a methyl or ethyl group, respectively, in proximity to the hydrazone bond [31].

To further analyze their structure-activity relationships, in the present study, we designed and synthesized derivatives of SIH, HAPI and HPPI (Fig. 1). The first modification was the reduction of the hydrazone bond leading to *N′*-(2-hydroxybenzyl)isonicotinoylhydrazide (redSIH; Fig. 1), *N′*-[1-(2-hydroxyphenyl)ethyl]isonicotinoylhydrazide (redHAPI; Fig. 1) and *N′*-[1-(2-hydroxyphenyl)propyl]isonicotinoylhydrazide (redHPPI; Fig. 1). These compounds were specifically synthesized to assess the importance of the hydrazone bond for the anti-oxidative and/or cytotoxic activity that has been associated with various aroylhydrazones [34], [35], [36].

We also studied the effects of bromination at position 5 of the phenolic ring of HAPI and HPPI, leading to (*E*)-*N′*-[1-(5-bromo-2-hydroxyphenyl)ethylidene]isonicotinoylhydrazide (BHAPI; Fig. 1) and (*E*)-*N′*-[1-(5-bromo-2-hydroxyphenyl)propylidene]isonicotinoylhydrazide (BHPPI; Fig. 1), respectively. The effect of halogenation was examined since a previous study demonstrated the high cytotoxic activity of a chloro-substituted ligand [31]. An analog, in which the phenolic ring of HAPI was exchanged for pyridine, was also prepared ((*E*)-*N′*-[1-(pyridin-2-yl)ethylidene]isonicotinoylhydrazide; 2API; Fig. 1) to assess the effects on its properties of changing the ligating groups from *O*, *N*, *O* to *N*, *N*, *O*. The chelation properties of 2API would be different from those of SIH because the pyridine nitrogen in 2API is a softer base relative to the hard phenolic oxygen in SIH. Considering this, 2API would be able to bind both Fe^3+^ and Fe^2+^ ions, while SIH and its analogs prefer Fe^3+^. Thus, although 2API does not belong to the same ligand category as SIH, we aimed to explore its properties because 2API is a methyl analog of 2-pyridylcarboxaldehyde isonicotinoyl hydrazone (PCIH), which was developed for the treatment of iron overload [37], [38].

An analog of HAPI with a 2C side chain as a part of an indane ring was also synthesized, leading to (*E*)-*N′*-(7-hydroxy-2,3-dihydro-1*H*-inden-1-ylidene)isonicotinoylhydrazide (7HII; Fig. 1). Analogs of HAPI and HPPI with varying alkyl groups adjacent to the hydrazone bond were also prepared, including derivatives containing an isopropyl substituent ((*E*)-*N′*-(1-(2-hydroxyphenyl)-2-methylpropylidene)isonicotinoylhydrazide; H16; Fig. 1), propyl substituent ((*E*)-*N′*-[1-(2-hydroxyphenyl)butylidene]isonicotinoylhydrazide; H17; Fig. 1), isobutyl substituent ((*E*)-*N′*-(1-(2-hydroxyphenyl)-3-methylbutylidene)isonicotinoylhydrazide; H18; Fig. 1), or cyclohexyl ring ((*E*)-*N′*-[cyclohexyl(2-hydroxyphenyl)methylene]isonicotinoylhydrazide; H28; Fig. 1).

To characterize these new ligands, we examined their: (***1***) stability against hydrolysis in plasma; (***2***) iron chelation and redox properties; (***3***) protective potential against oxidative injury induced by exposure of H9c2 rat embryonic cardiomyoblast cells to hydrogen peroxide (H_2_O_2_); (***4***) cytotoxic activity using neoplastic MCF-7 breast adenocarcinoma cells; and (***5***) selectivity by comparing their cytotoxic effects to the non-tumorigenic, cardiomyoblast cell line, H9c2. These studies are important for dissecting structure-activity relationships that are essential for the development of more effective ligands.

## Materials and Methods

### 1 Syntheses of chelators

All chemicals were purchased from Sigma-Aldrich (St. Louis, MO, USA). Thin layer chromatography was performed on TLC sheets (silica gel 60 F254) from Merck (Darmstadt, Germany). Microwave reactions were conducted in a Milestone Micro-SYNTH Ethos 1600 URM apparatus. Melting points were measured on a Kofler apparatus and are uncorrected. All products were characterized by NMR (Varian Mercury Vx BB 300 or VNMR S500 NMR spectrometers). Chemical shifts were reported as δ values in parts per million (ppm) and were indirectly referenced to tetramethylsilane (TMS) *via* the solvent signal. All assignments were based on 1D experiments. Elemental analysis was measured on a CHNS-OCE FISONS EA 1110 apparatus.

#### *N′-*Salicylaldehyde isonicotinoyl hydrazone (SIH)

SIH was synthesized as described previously [39]. Yellow crystalline solid. mp 232–234°C. ^1^H NMR (300 MHz, DMSO-*d_6_*): *δ* 12.29 (s, 1H, OH), 11.02 (s, 1H, NH), 8.80 (d, *J* = 4.4 Hz, 2H, Py), 8.68 (s, 1H, CH), 7.85 (d, *J* = 4.4 Hz, 2H, Py), 7.61 (dd, *J* = 7.7, 1.5 Hz, 1H, Ph), 7.36–7.28 (m, 1H, Ph), 6.95–6.88 (m, 2H, Ph). ^13^C NMR (75 MHz, DMSO-*d_6_*): *δ* 163.1, 157.5, 150.4, 148.9, 141.2, 131.7, 129.2, 121.5, 119.5, 116.4.

#### *N′*-(2-hydroxybenzyl)isonicotinoylhydrazide (redSIH)

SIH (0.69 g, 2.8 mmol) was dissolved in 96% (v/v) ethanol (50 mL) and NaBH_3_CN (0.36 g, 5.7 mmol) was added. The reaction mixture was adjusted to a pH of 3-5 using a 10% (v/v) solution of HCl in methanol. The reaction mixture was stirred at room temperature (RT) overnight and was then neutralized with a solution of sodium bicarbonate to pH 7. The reaction mixture was evaporated to dryness and was then partitioned against water and EtOAc. The combined organic layers were dried with anhydrous Na_2_SO_4_ and evaporated under reduced pressure. The product was purified with column chromatography on silica using hexane/EtOAc (1∶1) as a mobile phase. The product was isolated as a white crystalline solid. Yield 0.17 g (24%). mp 143–146°C. ^1^H NMR (300 MHz, DMSO-*d_6_*): *δ* 10.43 (s, 1H, OH), 9.61 (s, 1H, NH), 8.70 (d, *J* = 5.1 Hz, 2H, Py), 7.74–7.65 (m, 2H, Py), 7.20 (dd, *J* = 7.5, 1.7 Hz, 1H, Ph), 7.12–7.02 (m, 1H, Ph), 6.84–6.67 (m, 2H, Ph), 5.65 (s, 1H, NH), 3.95 (d, *J* = 6.0 Hz, 2H, CH_2_). ^13^C NMR (75 MHz, DMSO-*d_6_*): *δ* 163.5, 156.1, 150.4, 140.3, 130.1, 128.5, 124.1, 121.3, 118.9, 115.3, 50.7. Anal. Calcd. for C_13_H_13_N_3_O_2_: C, 64.19; H, 5.39; N, 17.27; Found: C, 64.50; H, 5.26; N, 17.56.

#### *N′*-[1-(2-Hydroxyphenyl)ethyl]isonicotinoylhydrazide (redHAPI)

The initial chelator, (*E*)-*N′*-[1-(2-hydroxyphenyl)ethylidene]isonicotinoylhydrazide (HAPI), was synthesized as described previously [33]. The reduced analog, redHAPI, was prepared from HAPI as described above for redSIH. The product was isolated as a yellow solid. Yield 0.18 g (26%). mp 131–134°C. ^1^H NMR (500 MHz, DMSO-*d_6_*): *δ* 10.29 (s, 1H, OH), 9.63 (s, 1H, NH), 8.70 (m, 2H, Py), 7.68 (d, *J* = 4.8 Hz, 2H, Py), 7.29 (dd, *J* = 7.8, 1.7 Hz, 1H, Ph), 7.11–7.00 (m, 1H, Ph), 6.82–6.69 (m, 2H, Ph), 5.57 (s, 1H, NH), 4.53–4.27 (m, 1H, CH), 1.28 (d, *J* = 6.6 Hz, 3H, CH_3_). ^13^C NMR (125 MHz, DMSO-*d_6_*): *δ* 164.1, 155.4, 150.4, 140.3, 129.1, 127.9, 127.3, 124.9, 119.1, 115.6, 54.3, 19.7. Anal. Calcd. for C_14_H_15_N_3_O_2_: C, 65.36; H, 5.88; N, 16.33; Found: C, 64.98; H, 6.04; N, 16.53.

#### *N′*-[1-(2-Hydroxyphenyl)propyl]isonicotinoylhydrazide (redHPPI)

The initial chelator, (*E*)-*N′*-[1-(2-hydroxyphenyl)propylidene]isonicotinoylhydrazide (HPPI), was synthesized as described previously [33]. The reduced analog, redHPPI was prepared from HPPI as described above for redSIH. The product was obtained as a yellow solid. Yield 0.29 g (42%). mp 115–118°C. ^1^H NMR (500 MHz, DMSO-*d_6_*): *δ* 10.21 (s, 1H, NH), 9.58 (s, 1H, NH), 8.90–8.55 (m, 2H, Py), 7.83–7.55 (m, 2H, Py), 7.27 (dd, *J* = 7.6, 1.7 Hz, 1H, Ph), 7.10 (m, 1H, Ph), 6.82–6.68 (m, 2H, Py), 5.62 (s, 1H, OH), 4.21 (t, *J* = 6.6 Hz, 1H, CH), 1.81–1.60 (m, 2H, CH_2_), 1.14 (t, *J* = 7.6 Hz, 3H, CH_3_). ^13^C NMR (125 MHz, DMSO-*d_6_*): *δ* 163.8, 155.8, 150.3, 140.3, 128.4, 127.8, 124.9, 118.9, 115.6, 60.6, 26.0, 10.7. Anal. Calcd. for C_15_H_17_N_3_O_2_: C, 66.40; H, 6.32; N, 15.49; Found: C, 66.42; H, 6.45; N, 15.55.

#### (*E*)-*N′*-[1-(5-Bromo-2-hydroxyphenyl)ethylidene]isonicotinoylhydrazide (BHAPI)

Isoniazid (0.21 g, 1.5 mmol), 5-bromo-2-hydroxyacetophenone (0.32 g, 1.5 mmol) and acetic acid (0.25 mL) were dissolved in methanol (5 mL) and stirred for 2 h under reflux in the microwave reactor described above. The reaction mixture was then cooled to 4°C and the resulting precipitate was collected by filtration, washed with water and methanol and dried over P_2_O_5_ to give 0.2 g (39%) of the product as a yellow crystalline solid. mp 225–227°C. ^1^H NMR (500 MHz, DMSO-*d_6_*): *δ* 13.27 (s, 1H, OH), 11.66 (s, 1H, NH), 8.82–8.76 (m, 2H, Py), 7.86–7.79 (m, 2H, Py), 7.76 (d, *J* = 2.3 Hz, 1H, Ph), 7.45 (dd, *J* = 8.8, 2.2 Hz, 1H, Ph), 6.90 (d, *J* = 8.7 Hz, 1H, Ph), 2.49 (s, 3H, CH_3_). ^13^C NMR (125 MHz, DMSO-*d_6_*): *δ* 163.3, 158.1, 158.0, 150.4, 140.1, 134.1, 131.0, 122.2, 121.5, 119.8, 109.9, 14.7. Anal. Calcd. for C_14_H_14_BrN_3_O_2_: C, 50.32; H, 3.62; N, 12.57; Found: C 50.71; H, 3.99; N, 12.88.

#### (*E*)-*N′*-[1-(5-Bromo-2-hydroxyphenyl)propylidene]isonicotinoylhydrazide (BHPPI)

Isoniazid (0.2 g, 1.4 mmol), 5-bromo-2-hydroxypropiophenone (0.33 g, 1.4 mmol) and acetic acid (0.25 mL) were dissolved in methanol (5 mL) and stirred overnight under reflux. After cooling the reaction mixture to 4°C, the resulting precipitate was collected by filtration, washed with water and methanol and dried over P_2_O_5_ to give 0.32 g (64%) of the product as a yellow crystalline solid. mp 239–242°C. ^1^H NMR (500 MHz, DMSO-*d_6_*): *δ* 13.33 (s, 1H, OH), 11.69 (s, 1H, NH), 8.79 (d, *J* = 5.0 Hz, 2H, Py), 7.86–7.81 (m, 3H, Py, Ph), 7.45 (dd, *J* = 8.8, 2.4 Hz, 1H, Ph), 6.92 (d, *J* = 8.7 Hz, 1H, Ph), 3.01 (q, *J* = 7.6 Hz, 2H, CH_2_), 1.09 (t, *J* = 7.0 Hz, 3H, CH_3_). ^13^C NMR (125 MHz, DMSO-*d_6_*): *δ* 163.8, 161.2, 158.5, 150.3, 140.3, 134.0, 130.5, 122.4, 120.2, 120.1, 109.9, 19.7, 11.4. Anal. Calcd. for: C_15_H_16_BrN_3_O_2_: C, 51.74; H, 4.05; N, 12.07; Found: C, 51.41; H, 4.26; N, 11.74.

#### (*E*)-*N′*-[1-(Pyridin-2-yl)ethylidene]isonicotinoylhydrazide (2API)

Isoniazid (0.57 g, 4.1 mmol), 2-acetylpyridine (0.5 g, 3.4 mmol) and acetic acid (0.25 mL) were dissolved in methanol (10 mL) and stirred overnight under reflux. After cooling the reaction mixture to 4°C, the resulting precipitate was collected by filtration, washed with water and methanol and dried over P_2_O_5_ to give 0.37 g (37%) of the product as a white crystalline solid. mp 166°C. ^1^H NMR (500 MHz, DMSO-*d_6_*): *δ* 11.10 (s, 1H, NH), 8.77 (d, *J* = 5.1 Hz, 2H, Py), 8.62 (d, *J* = 5.3 Hz, 1H, Py'), 8.12 (d, *J* = 8.2 Hz, 1H, Py'), 7.90–7.40 (m, 1H, Py'), 7.83–7.77 (m, 2H, Py), 7.47–7.40 (m, 1H, Py'), 2.47 (s, 3H, CH_3_). ^13^C NMR (125 MHz, DMSO-*d_6_*): *δ* 163.0, 155.1, 150.3, 149.7, 141.2, 136.9, 124.7, 122.2, 121.2, 13.1. Anal. Calcd. for C_13_H_12_N_4_O: C, 64.99; H, 5.03; N, 23.32; Found: C, 65.33; H, 5.31; N, 23.43.

#### (*E*)-*N′*-(7-Hydroxy-2,3-dihydro-1*H*-inden-1-ylidene)isonicotinoylhydrazide (7HII)

Isoniazid (0.46 g, 3.4 mmol), 7-hydroxy-2,3-dihydro-1*H*-inden-1-one (0.5 g, 3.4 mmol) and acetic acid (0.25 mL) were dissolved in methanol (10 mL) and stirred overnight under reflux. After cooling the reaction mixture to 4°C, the resulting precipitate was collected by filtration and washed with water and methanol. The solid was suspended in toluene and was stirred for 30 min. This solution was filtered to obtain 0.37 g (66%) of the product as a yellow crystalline solid. mp 232–236°C. ^1^H NMR (500 MHz, DMSO-*d_6_*): *δ* 11.30 (s, 1H, NH), 10.15 (s, 1H, OH), 8.80–8.70 (m, 2H, Py), 7.84–7.74 (m, 2H, Py), 7.30 (dd, *J* = 7.8, 1.3 Hz, 1H, Ph), 6.89 (d, *J* = 7.4 Hz, 1H, Ph), 6.75 (d, *J* = 8.1 Hz, 1H, Ph), 3.13–3.00 (m, 4H, 2xCH_2_). ^13^C NMR (125 MHz, DMSO-*d_6_*): *δ* 167.9, 162.5, 155.5, 150.3, 150.0, 140.8, 133.1, 122.6, 122.1, 116.6, 113.0, 28.7, 28.1. Anal. Calcd. for C_15_H_13_N_3_O_2_: C, 67.40; H, 4.90; N, 15.72; Found: C, 67.02; H, 5.13; N, 15.87.

#### (*E*)-*N′*-(1-(2-Hydroxyphenyl)-2-methylpropylidene)isonicotinoylhydrazide (H16)

To prepare H16, 1-(2-hydroxyphenyl)-2-methylpropan-1-one was first synthesized: 2-hydroxybenzonitrile (0.36 g, 3 mmol) was dissolved in dry THF (5 mL) and a solution of isopropylmagnesium chloride in THF (2 M, 6.1 mL, 1.2 mmol) was added and the reaction mixture refluxed for 2 h. The reaction mixture was cooled in an ice bath, 10 mL of cold water was carefully added and cold concentrated H_2_SO_4_ added dropwise to obtain an acidic pH. The reaction mixture was then heated for 1 h at 80°C and, after cooling to RT, it was extracted twice with diethyl ether. The combined organic layer was dried with anhydrous Na_2_SO_4_ and evaporated under reduced pressure. The product was purified by column chromatography on silica using hexane/EtOAc (40∶1) as the mobile phase. The product was obtained as a yellow oil. Yield 0.45 g (91%). ^1^H NMR (300 MHz, CDCl_3_): *δ* 12.52 (s, 1H, OH), 7.79 (dd, *J* = 8.1, 1.6 Hz, 1H, Ph), 7.53–7.40 (m, 1H, Ph), 7.04–6.84 (m, 2H, Ph), 3.70–3.54 (m, 1H, CH), 1.43–1.14 (m, 6H, 2xCH_3_). ^13^C NMR (75 MHz, CDCl_3_): *δ* 210.9, 163.1, 136.2, 129.8, 118.8, 118.7, 118.1, 34.9, 29.7, 19.3.

1-(2-Hydroxyphenyl)-2-methylpropan-1-one (0.29 g, 1.8 mmol), isoniazid (0.24 g, 1.8 mmol) and acetic acid (0.25 mL) were dissolved in methanol (5 mL) and heated at 110°C in an autoclave for 48 h. After cooling to RT, water was added dropwise until the solution turned cloudy and the mixture was left to crystallize at 4°C for 24 h. The precipitate was collected by filtration, washed with water and methanol and dried over P_2_O_5_ to yield 0.07 g (14%) of H16 as a white crystalline solid. mp 228–235°C. ^1^H NMR (300 MHz, DMSO-*d_6_*): *δ* 10.08 (s, 1H, NH), 8.66 (m, 2H, Py), 7.66 (m, 2H, Py), 7.43 (d, *J* = 7.7 Hz, 1H, Ph), 7.35–7.22 (m, 1H, Ph), 7.13 (d, *J* = 7.7 Hz, 1H, Ph), 7.03–6.79 (m, 1H, Ph), 3.07–2.80 (m, 1H, CH), 1.42–0.78 (m, 6H, 2xCH_3_). ^13^C NMR (75 MHz, DMSO-*d_6_*): *δ* 163.7, 163.3, 153.9, 150.4, 141.4, 130.9, 128.8, 121.2, 120.7, 119.7, 116.2, 35.9, 20.1. Anal. Calcd. for C_16_H_17_N_3_O_2_: C, 67.83; H, 6.05; N, 14.83; Found: C, 67.44; H, 6.37; N, 14.83.

#### (*E*)-*N′*-[1-(2-Hydroxyphenyl)butylidene]isonicotinoylhydrazide (H17)

To prepare H17, 1-(2-hydroxyphenyl)butan-1-one was first synthesized: Magnesium (0.3 g, 12 mmol) was suspended in dry THF (5 mL), and propylbromide (1.5 g, 12 mmol) was added dropwise and the mixture refluxed for 2 h until the magnesium dissolved. After cooling the reaction mixture to RT, 2-hydroxybenzonitrile (0.36 g, 3 mmol) dissolved in dry THF (5 mL) was added dropwise and the reaction refluxed for 2 h. The reaction mixture was cooled in an ice bath, 10 mL of cold water was carefully added and cold concentrated H_2_SO_4_ added dropwise to obtain an acidic pH. The reaction mixture was then heated for 1 h at 80°C and, after cooling to RT, it was extracted twice with diethyl ether. The combined organic layer was dried with anhydrous Na_2_SO_4_ and evaporated under reduced pressure. The product was purified by column chromatography on silica (gradient; hexane to hexane/EtOAc 40∶1). The product was obtained as a yellow oil. Yield: 0.41 g (82%). ^1^H NMR (300 MHz, CDCl_3_): *δ* 7.90–7.67 (m, 1H, Ph), 7.57–7.36 (m, 1H, Ph), 7.05–6.76 (m, 2H, Ph), 2.79 (t, *J* = 7.3 Hz, 2H, CH_2_), 1.69–1.43 (m, 2H, CH_2_), 1.03 (t, *J* = 7.4 Hz, 3H, CH_3_). ^13^C NMR (75 MHz, CDCl_3_): *δ* 206.8, 162.5, 136.2, 130.0, 119.4, 118.8, 118.5, 40.2, 17.9, 13.8.

1-(2-Hydroxyphenyl)-butan-1-one (0.39 g, 2.4 mmol), isoniazid (0.33 g, 2.4 mmol) and acetic acid (0.25 mL) were dissolved in methanol (5 mL) and heated at 110°C in an autoclave for 72 h. After cooling to RT, water was added dropwise until the solution turned cloudy and the mixture was left to crystallize at 4°C for 24 h. The precipitate was collected by filtration, washed with water and methanol and dried over P_2_O_5_ to yield 0.08 g (12%) of the product as a white crystalline solid. mp 189–192°C. ^1^H NMR (500 MHz, DMSO-*d_6_*): *δ* 8.79 (d, *J* = 5.3, 2H, Py), 7.89–7.72 (m, 2H, Py), 7.63 (dd, *J* = 8.1, 1.6 Hz, 1H, Ph), 7.30 (ddd, *J* = 8.4, 7.1, 1.5 Hz, 1H, Ph), 7.00–6.79 (m, 2H, Ph), 3.06–2.86 (m, 2H, CH_2_), 1.73–1.44 (m, 2H, CH_2_), 0.99 (t, *J* = 7.3 Hz, 3H, CH_3_). ^13^C NMR (125 MHz, DMSO-*d_6_*): *δ* 163.6, 161.7, 159.4, 150.3, 140.5, 131.6, 128.7, 122.3, 118.8, 118.3, 117.8, 27.9, 20.3, 13.9. Anal. Calcd. for C_16_H_17_N_3_O_2_: C, 67.83; H, 6.05; N, 14.83; Found: C, 67.42; H, 6.41; N, 14.55.

#### (*E*)-*N′*-(1-(2-Hydroxyphenyl)-3-methylbutylidene)isonicotinoylhydrazide (H18)

To prepare H18, 1-(2-hydroxyphenyl)-3-methylbutan-1-one was first synthesized: Magnesium (0.41 g, 16.9 mmol) was suspended in dry THF (5 mL), and isobutylbromide (2.31 g, 16.7 mmol) was added dropwise and the mixture refluxed for 2 h until the magnesium dissolved. After cooling the reaction mixture to RT, 2-hydroxybenzonitrile (0.33 g, 2.8 mmol) dissolved in dry THF (5 mL) was added dropwise and the reaction refluxed for 2 h. The reaction mixture was cooled in an ice bath, 10 mL of cold water was carefully added and cold concentrated H_2_SO_4_ added dropwise to obtain an acidic pH. The reaction mixture was then heated for 1 h at 80°C and, after cooling to RT, it was extracted twice with diethyl ether. The combined organic layer was dried with anhydrous Na_2_SO_4_ and evaporated under reduced pressure. The product was purified by column chromatography on silica using hexane/EtOAc (40∶1) as the mobile phase. The product was a yellow oil. Yield 0.45 g (91%). ^1^H NMR (300 MHz, CDCl_3_): *δ* 12.48 (s, 1H, OH), 7.89–7.63 (m, 1H, Ph), 7.59–7.40 (m, 1H, Ph), 7.08–6.83 (m, 2H, Ph), 2.92–2.74 (m, 2H, CH_2_), 2.38–2.22 (m, 1H, CH), 1.13–0.94 (m, 6H, 2xCH_3_). ^13^C NMR (75 MHz, CDCl_3_): *δ* 206.7, 162.6, 136.2, 130.1, 119.6, 118.8, 118.5, 47.1, 25.5, 22.7.

1-(2-Hydroxyphenyl)-3-methylbutan-1-one (0.2 g, 1.1 mmol), isoniazid (0.12 g, 1.1 mmol) and acetic acid (0.25 mL) were dissolved in methanol (5 mL) and heated at 110°C in an autoclave for 72 h. After cooling to RT, water was added dropwise until the solution turned cloudy and the mixture was left to crystallize at 4°C. The precipitate was then collected by filtration, washed with water and methanol and dried over P_2_O_5_. The overall yield of the white crystalline product was 0.06 g (19%). mp 144–146°C. ^1^H NMR (500 MHz, DMSO-*d_6_*): *δ* 13.27 (s, 1H, OH), 11.61 (s, 1H, NH), 8.82–8.77 (m, 2H, Py), 7.81–7.73 (m, 2H, Py), 7.65 (dd, *J* = 8.0, 1.7 Hz, 1H, Ph), 7.42–7.19 (m, 1H, Ph), 6.97–6.86 (m, 2H, Ph), 2.99 (d, *J* = 7.4 Hz, 2H, CH_2_), 2.02–1.93 (m, 1H, CH), 0.95 (d, *J* = 6.6 Hz, 6H, 2xCH_3_). ^13^C NMR (125 MHz, DMSO-*d_6_*): *δ* 163.3, 161.5, 159.2, 150.4, 140.4, 131.6, 129.0, 122.2, 118.7, 117.8, 34.3, 27.6, 22.2. Anal. Calcd. for C_17_H_19_N_3_O_2_: C, 68.67; H, 6.44; N, 14.13; Found: C, 68.28; H, 6.69; N, 13.85.

#### (*E*)-*N′*-[Cyclohexyl(2-hydroxyphenyl)methylene]isonicotinoylhydrazide (H28)

To prepare H28, cyclohexyl(2-hydroxyphenyl)methanone was synthesized: Magnesium (0.36 g, 15 mmol) was suspended in dry THF (5 mL) and cyclohexylbromide (2.4 g, 15 mmol) was added dropwise. This reaction mixture was refluxed for 2 h until the magnesium dissolved. After cooling the reaction mixture to RT, 2-hydroxybenzonitrile (0.29 g, 2.4 mmol) dissolved in dry THF (5 mL) was added dropwise and the reaction refluxed for 3 h. The reaction mixture was cooled in an ice bath, 10 mL of cold water was added carefully and then cold concentrated H_2_SO_4_ was added dropwise to obtain an acidic pH. The reaction mixture was then heated overnight at 80°C and, after cooling to RT, it was extracted twice with diethyl ether. The combined organic layer was dried with anhydrous Na_2_SO_4_ and evaporated under reduced pressure. The product was purified by column chromatography on silica (gradient; hexane to hexane/EtOAc 40∶1). The product was obtained as a yellow oil. Yield 0.49 g (97%). ^1^H NMR (300 MHz, CDCl_3_) *δ* 12.58 (s, 1H, OH), 7.88–7.69 (m, 1H, Ph), 7.55–7.32 (m, 1H, Ph), 7.07–6.95 (m, 2H, Ph), 3.43–3.12 (m, 1H, Cy), 2.03–1.06 (m, 10H, Cy). ^13^C NMR (75 MHz, CDCl_3_): *δ* 210.1, 163.1, 136.1, 129.8, 118.7, 118.7, 118.2, 45.2, 29.5, 25.8, 25.7.

Cyclohexyl(2-hydroxyphenyl)methanone (0.19 g, 0.93 mmol), isoniazid (0.13 g, 0.93 mmol) and acetic acid (0.20 mL) were dissolved in methanol (5 mL) and heated at 110°C in an autoclave for 4 days. After cooling to RT, water was added dropwise until the solution turned cloudy and the mixture was left to crystallize at 4°C for 24 h. The precipitate was collected by filtration, washed with methanol and dried over P_2_O_5_ to give 0.046 g (15%) of the product as a white solid. mp 251–253°C. ^1^H NMR (300 MHz, DMSO-*d_6_*): *δ* 9.27 (s, 1H, NH), 8.70–8.63 (m, 2H, Py), 7.86–7.70 (m, 2H, Py), 7.51–7.34 (m, 1H, Ph), 7.33–7.19 (m, 1H, Ph), 7.17–7.02 (m, 1H, Ph), 7.02–6.78 (m, 1H, Ph), 2.69–2.34 (m, 1H, Cy), 1.92–1.00 (m, 10H, Cy). ^13^C NMR (75 MHz, DMSO-*d_6_*): *δ* 163.0, 161.3, 150.4, 141.4, 130.8, 128.8, 121.2, 120.8, 119.7, 116.2, 30.3, 29.9, 26.0, 25.8. Anal. Calcd. for C_19_H_21_N_3_O_2_: C, 70.57; H, 6.55; N, 12.99; Found: C, 70.36; H, 6.91; N, 13.03.

### 2 Stability study

#### 2.1 HPLC instrument and chromatographic conditions

HPLC analyses were performed on a Prominence LC 20A chromatographic system (Shimadzu, Kyoto, Japan) consisting of a DGU-20A3 degasser, two LC-20AD pumps, SIL-20AC autosampler, a CTO-20AC column oven, SPD-20AC detector and a CBM-20AC communication module. The data were processed by LC solution software, version 1.21 SP1 (Shimadzu).

Analysis of new chelators were performed using an Ascentis C18 chromatographic column (10×3 mm, 3 µm) protected with a guard column with the same sorbent (Sigma-Aldrich). The mobile phase was composed of 1 mM EDTA in 5 mM phosphate buffer and methanol in different ratios (Table 1). The column oven was set at 25°C and the autosampler at 5°C. A flow rate of 0.3 mL/min and injection volume of 20 µL were used. Chromatographic conditions for the determination of each chelator are given in Table 1.

**Table 1:** Chromatographic conditions used for the determination of the stability of the new chelators in rabbit plasma.

Chelator	Mobile phase ratio (v/v)	UV (nm)	IS
**redSIH**	40∶60	254	redHAPI
**redHAPI**	40∶60	254	7HII
**redHPPI**	40∶60	254	7HII
**BHAPI**	30∶70	254	o-108
**BHPPI**	30∶70	254	7HII
**2API**	40∶60	297	SIH
**7HII**	30∶70	297	BHPPI
**H16**	40∶60	254	o-108
**H17**	30∶70	297	H28
**H18**	30∶70	297	H28
**H28**	30∶70	254	H18

The linearity, precision and accuracy of the methods were examined by the analysis of plasma samples spiked with different amounts of the chelators. Selectivity was confirmed by an analysis of blank plasma samples. All evaluated parameters reached acceptable values [40]. SIH was analyzed using a previously developed and validated method [41].

#### 2.2 Assessment of the chelator stabilities in rabbit plasma

The drug-free plasma samples were spiked with a standard solution of each chelator (1 mg/mL in DMSO) to obtain a concentration of 100 µM. The final chelator-spiked plasma samples were maintained at 37°C and stirred at 300 rpm. Samples of the studied chelators in plasma (50 µL) were transferred into Eppendorf tubes on ice at time intervals of *t* = 0, 60, 120, 180, 240, 300, 360, 420, 480, 540 and 600 min from the beginning of the experiment. After this procedure, internal standards (IS) were added to the samples and then the plasma proteins were precipitated by adding methanol (200 µL). Precipitates were separated by centrifugation (10,000 rpm/10 min) and the clear supernatant was injected onto the column. In the case of redSIH, redHAPI, redHPPI and 2API, the supernatant was diluted using deionized water at a 1∶1 ratio to obtain acceptable peak shapes.

### 3 Biological studies

#### 3.1 Chemicals

Constituents for various buffers as well as other chemicals (*e.g.*, various iron salts) were purchased from Sigma-Aldrich, Merck or Penta (Prague, Czech Republic) and were of the highest pharmaceutical or analytical grade available.

#### 3.2 Cell cultures

The MCF-7 human breast adenocarcinoma cell line was purchased from the European Collection of Cell Cultures (ECACC; Salisbury, UK), and the H9c2 cardiomyoblast cell line, derived from embryonic rat heart tissue, was obtained from the American Type Culture Collection (ATCC; Manassas, VA, USA). Cells were cultured in Dulbecco's modified Eagle's medium (DMEM; Lonza, Verviers, Belgium) with (H9c2) or without (MCF-7) phenol red and were supplemented with 10% (v/v) heat-inactivated fetal bovine serum (FBS; Lonza), 1% penicillin/streptomycin solution (Lonza) and 10 mM HEPES buffer (pH 7.0–7.6; Sigma-Aldrich). Both cell lines were cultured in 75 cm^2^ tissue culture flasks (TPP, Trasadingen, Switzerland) at 37°C in a humidified atmosphere of 5% CO_2_. Sub-confluent cells (70–80% confluency) were sub-cultured every 3-4 days.

#### 3.3 Determination of iron chelating efficacy in solution

To assess the iron chelation efficiency of the newly synthesized agents in solution, their ability to remove iron from the iron-calcein complex was examined [42]. Calcein is a fluorescent probe that readily forms iron complexes [42]. Upon formation of the iron-calcein complex, the fluorescence of calcein is quenched. The addition of another chelating agent to the iron-calcein complex leads to the removal of iron from this complex, resulting in the formation of the new iron-chelator complex. The removal of iron from the iron-calcein complex is accompanied by an increase in fluorescence intensity (*i.e.*, de-quenching), due to the formation of free calcein. Thus, the measurement of calcein fluorescence intensity was used to examine the iron chelation efficacy of the novel chelators [42].

A complex of calcein (free acid, 20 nM; Molecular Probes, Eugene, OR, USA) with iron derived from ferrous ammonium sulfate (200 nM) was prepared in HBS buffer (150 mM NaCl, 40 mM HEPES, pH 7.2). Calcein and ferrous ammonium sulfate were continuously stirred for 45 min in the dark, after which >90% of the fluorescence was quenched. Then, 995 µL of the complex was pipetted into a stirred cuvette and baseline measurements were acquired. After 100 s, 5 µL of the novel chelator solution was added, yielding a final chelator concentration of 5 µM. Fluorescence intensity change was measured as a function of time at RT using a Perkin Elmer LS50B fluorimeter (Perkin Elmer, Waltham, MA, USA) at λ_ex_ = 486 nm and λ_em_ = 517 nm for 350 s. The iron chelation efficiency in solution was expressed as a percentage of the efficiency of the reference chelator, SIH (100%).

#### 3.4 Calcein-AM assay to assess the cell membrane permeability and access to the labile iron pool

These experiments were performed according to Glickstein *et al.* [43] with slight modifications. MCF-7 cells were seeded in 96-well plates (10,000 cells per well). Cells were loaded with iron using the iron donor, ferric ammonium citrate (530 µg/mL), 24 h prior to the experiment, and the cells then washed. To prevent potential interference (especially with regard to various trace elements), the medium was replaced with the ADS buffer (prepared using Millipore water (18.2 MΩ/cm) supplemented with 116 mM NaCl, 5.3 mM KCl, 1 mM CaCl_2_, 1.2 mM MgSO_4_, 1.13 mM NaH_2_PO_4_, 5 mM d-glucose, and 20 mM HEPES, pH 7.4). Cells were then loaded with the membrane-permeant, calcein green acetoxymethyl ester (calcein-AM; 2 µM; Molecular Probes) for 30 min/37°C, and then washed. Cellular esterases cleave the acetoxymethyl groups to form the cell membrane-impermeant compound, calcein green, whose fluorescence is quenched upon binding iron. Intracellular fluorescence (λ_ex_ = 488 nm; λ_em_ = 530 nm) was then measured as a function of time (1 min before and 10 min after the addition of chelator) at 37°C using a Tecan Infinite 200 M plate reader (Tecan Group, Männedorf, Switzerland). The iron chelation efficiency in cells was expressed as a percentage of the efficiency of the reference chelator, SIH (100%).

#### 3.5 Preparation of ^59^Fe_2_-transferrin

Human Tf (Sigma-Aldrich) was labeled with Fe or ^59^Fe (PerkinElmer) to produce Fe_2_-Tf or ^59^Fe_2_-Tf, respectively, with a final specific activity of 500 pCi/pmol Fe, as previously described [34], [44]. Unbound ^59^Fe was removed by exhaustive vacuum dialysis against an excess of 0.15 M NaCl buffered at pH 7.4 with 1.4% (w/v) NaHCO_3_ by standard methods [34], [44].

#### 3.6 The effect of chelators on mobilizing cellular ^59^Fe

The ability of the novel ligands to mobilize ^59^Fe from MCF-7 cells was examined by conducting ^59^Fe efflux experiments using established techniques [34], [45]. In brief, after pre-labeling cells with ^59^Fe_2_-Tf (0.75 µM) for 3 h/37°C, the cell cultures were washed four times with ice-cold PBS and then subsequently incubated with each chelator (25 µM) for 3 h/37°C. The overlying media containing released ^59^Fe was then carefully separated from the cells using a Pasteur pipette. Radioactivity was measured in both the cell pellet and supernatant using a γ-scintillation counter (Wallac Wizard 3, Turku, Finland).

#### 3.7 The effect of the chelators on the prevention of cellular ^59^Fe uptake from ^59^Fe2-Tf

The ability of the chelators to prevent cellular ^59^Fe uptake from ^59^Fe_2_-Tf was examined using standard methods [46], [47]. In brief, MCF-7 cells were incubated with ^59^Fe_2_-Tf (0.75 µM) for 3 h/37°C in the presence of the assessed chelators (25 µM). The cells were then washed four times with ice-cold PBS and the internalized ^59^Fe was determined *via* established methods by incubating the cell monolayer for 30 min/4°C with the general protease, Pronase (1 mg/mL; Sigma-Aldrich). The cells were then removed from the monolayer with a plastic spatula and centrifuged for 1 min/12,000×*g*. The supernatant represents membrane-bound, Pronase-sensitive ^59^Fe that was released by the protease, while the Pronase-insensitive fraction represents internalized ^59^Fe [34], [46], [47]. The amount of internalized ^59^Fe was expressed as a percentage of the ^59^Fe internalized by untreated control cells (100%).

#### 3.8 Ascorbate oxidation assay for analysis of redox activity of iron complexes

The ability of the iron complexes of the novel ligands to mediate the oxidation of a physiological substrate, ascorbate, was examined using an established protocol [46], [48]. In brief, l-ascorbic acid (100 µM) was prepared immediately prior to the experiment and was incubated either alone or in the presence of Fe^3+^ (10 µM; as FeCl_3_) in a 50-fold molar excess (500 µM) of citrate and chelators (1-60 µM). Chelators were assayed at iron-binding equivalents (IBE) of 0.1 (excess of iron), 1 (iron-chelator complexes with a fully saturated coordination sphere) and 3 (excess of free chelator). The iron chelators, ethylenediaminetetraacetic acid (EDTA) and DFO, were used as positive and negative controls, respectively, as their redox activity has been well characterized [49]. The decrease in absorbance at 265 nm, which is the absorption maximum of ascorbate, was measured using the plate reader described previously after 10 and 40 min of incubation at RT. The decrease in absorbance between the two time points was calculated and expressed as a percentage of the control in the absence of the chelators (100%).

#### 3.9 Protection against oxidative injury and assessment of cytotoxicity

For these experiments, cells were seeded in 96-well plates (TPP) at a density of 10,000 cells/well (H9c2 rat cardiomyoblast) or 5,000 cells/well (MCF-7). H9c2 cells were seeded in the plates 48 h prior to addition of the studied ligands and 24 h prior to the experiments, the medium was changed to serum- and pyruvate-free DMEM (Sigma-Aldrich). The ability of the ligands to protect against oxidative injury was assessed by a simultaneous 24 h incubation with H_2_O_2_ (200 µM) in the presence and absence of varying concentrations of the chelators. The inherent cytotoxicity of the ligands was studied using the H9c2 cell line after a 72 h incubation. For proliferation studies, MCF-7 cells were seeded 24 h prior to addition of the chelators. The cytotoxic effects of the various iron chelators were then studied at different concentrations after a 72 h incubation. To dissolve the lipophilic agents, dimethyl sulfoxide (DMSO; Sigma-Aldrich) was utilized leading to a final DMSO concentration of 0.1% (v/v) in the culture medium of all groups. At this concentration, DMSO had no effect on cytotoxicity (data not shown). The viability of the H9c2 and MCF-7 cells was determined using the neutral red (NR; Sigma) uptake assay, which is based on the ability of viable cells to incorporate NR into lysosomes [33], [50]. The optical density of soluble NR was measured at λ = 540 nm using the Tecan Infinite 200 M plate reader. The viability or proliferation of the experimental groups was expressed as a percentage of the untreated controls (100%). Control experiments using viable cell counts demonstrated a direct correlation to NR uptake.

#### 3.10 Data analysis and statistics

The values of the molecular weights (MW) and n-octanol/water coefficients (log *P*_calc_; Table 2) were calculated using ChemBioOffice Ultra 11.0 software (CambridgeSoft, Cambridge, MA, USA). The log *P*_calc_ is expressed as an average of the results of Crippen's [51], Viswanadhan's [52], and Broto's [53] method. SigmaStat for Windows 3.5 (Systat Software, San Jose, CA, USA) statistical software was used for data analyses. The data are expressed as the mean ±S.D. of at least 3 experiments. Statistical significance was determined using a one-way ANOVA with a Bonferroni *post-hoc* test (comparisons of multiple groups against the relevant control). The results were considered to be statistically significant when *p*<0.05. The EC_50_ (half-maximal effective concentration) and IC_50_ (half-maximal inhibitory concentration) values were calculated using CalcuSyn 2.0 software (Biosoft, Cambridge, UK). Raw data underlying the findings in this study are in Data S1.

**Table 2:** Molecular weights (MW) and calculated n-octanol/water coefficients (log *P*_calc_) of the studied analogs.

Chelator	MW (g/mol)	log *P*_calc_
**SIH**	241	1.5
**redSIH**	243	1.0
**redHAPI**	257	1.4
**redHPPI**	271	1.9
**BHAPI**	334	2.1
**BHPPI**	348	2.6
**2API**	240	0.7
**7HII**	267	1.4
**H16**	283	2.2
**H17**	283	2.2
**H18**	297	2.5
**H28**	323	3.1

The MW and log *P*_calc_ values were calculated using ChemBioOffice Ultra 11.0 software. The log *P*_calc_ is expressed as an average of the results of Crippen's and Viswanadhan's fragmentations and Broto's method.

## Results

### 1 Stability of the chelators in plasma

**Figure 2:**
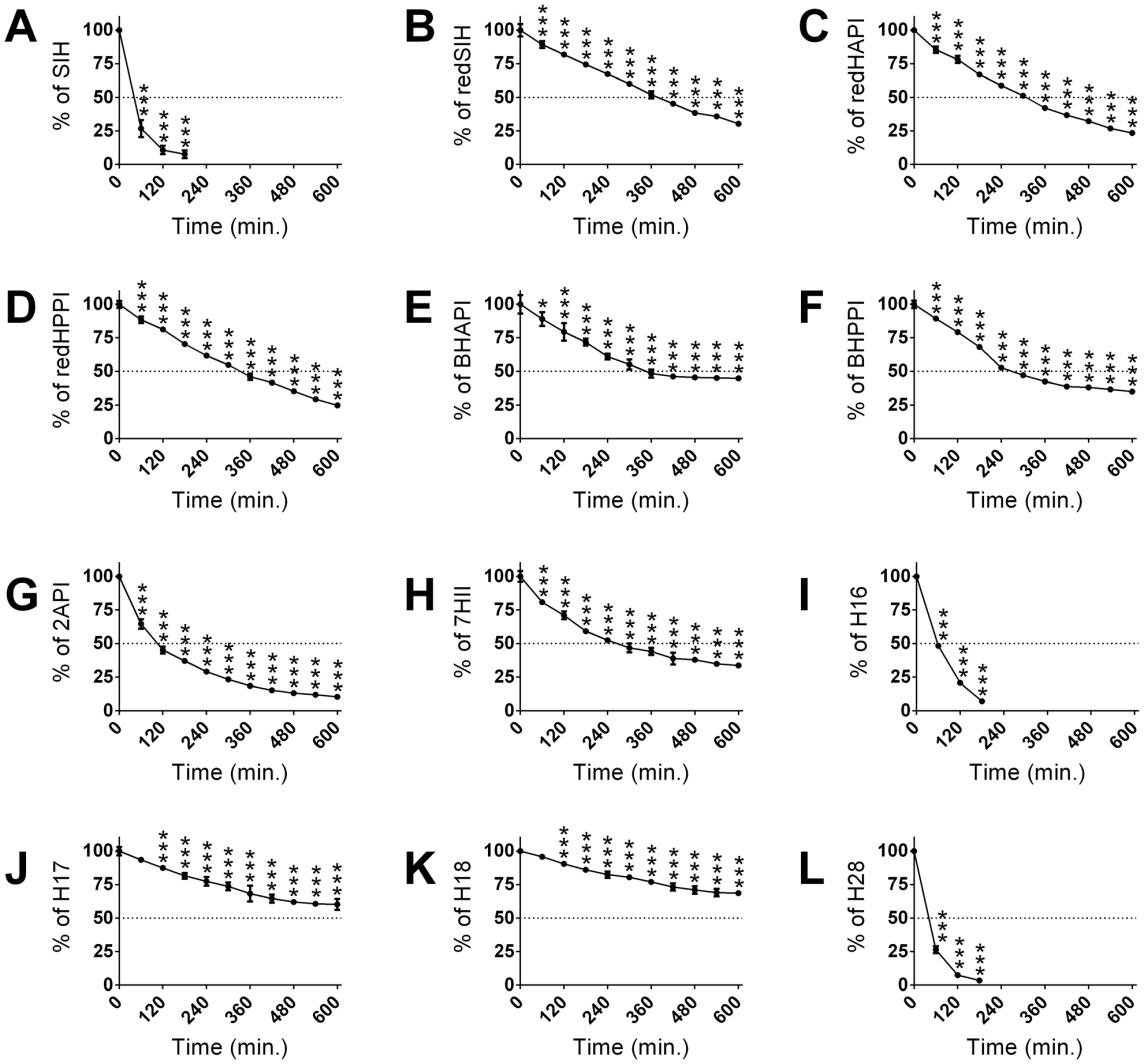
Stabilities of SIH and its novel analogs in rabbit plasma. SIH (**A**), redSIH (**B**), redHAPI (**C**), redHPPI (**D**), BHAPI (**E**), BHPPI (**F**), 2API (**G**), 7HII (**H**), H16 (**I**), H17 (**J**), H18 (**K**) and H28 (**L**) were incubated at 37°C in rabbit plasma and their concentrations were analyzed using HPLC every 60 min until *t* = 600 min. Results are expressed as a percentage of the concentration at *t* = 0 min (100 µM). Results are Mean ±SD (*n* = 3 experiments). Statistical significance (ANOVA): * *p*<0.05, ** *p*<0.01, *** *p*<0.001 compared to the concentration at *t* = 0 min (100%).

The stabilities of the newly prepared chelators in rabbit plasma were studied using HPLC analysis following a 600 min (10 h) incubation *in vitro*. The results were expressed as a percentage of the initial concentration of chelators at time *t* = 0 min. In our previous studies, SIH showed low stability, with less than 10% of SIH remaining intact after 180 min [33] and this was confirmed in our present investigation (Fig. 2A). The methylated and ethylated analogs of SIH, namely HAPI and HPPI, were markedly more resistant than SIH to hydrolysis in plasma. In fact, HAPI and HPPI were present at 26% and 41% of their original concentration at *t* = 600 min [33].

The reduction of the hydrazone bond of SIH caused a marked increase in the stability of redSIH, with 30% of the intact ligand remaining at *t* = 600 min (Fig. 2B). On the other hand, the reduction of the hydrazone bond of HAPI and HPPI, led to comparable or slightly decreased stability relative to SIH, with 23% of redHAPI and 25% of redHPPI remaining intact in plasma at *t* = 600 min (Fig. 2C, D). The bromination of HAPI increased the stability of BHAPI relative to SIH, with 45% of the ligand remaining intact, while the bromination of HPPI had no significant effect relative to SIH (*i.e.*, 35% of BHPPI remained intact at the end of the 600 min incubation period; Fig. 2E, F).

The 2-acetylpyridine derivative, 2API, showed better stability than the parent chelator, SIH, but was less stable than HAPI, with 10% of 2API remaining intact after the 600 min incubation (Fig. 2G). The cyclic ligand, 7HII, possessed comparable stability to HPPI, with 34% of 7HII remaining at the end of incubation (Fig. 2H). The introduction of a bulky isopropyl or cyclohexyl group to analogs H16 and H28, respectively, resulted in a surprisingly short half-life in plasma, with almost complete decomposition of these ligands at *t* = 180 min (Fig. 2I, L). Pilot experiments showed that the rapid decomposition of H16 was only partially due to hydrolysis of the hydrazone bond (only 10% of the expected ketone was found in plasma), with the instability probably also involving the hydrazine bond. Nevertheless, this remains to be carefully elucidated by using additional advanced analytical methods. In contrast, the introduction of an unbranched propyl or terminally-branched isobutyl moiety (ligands H17 and H18, respectively) led to a pronounced increase of their stability in plasma relative to SIH (Fig. 2 J, K), with 60% of H17 and 69% of H18 remaining intact in plasma after a 10 h incubation.

### 2. Determination of the iron chelating efficacy in solution and in MCF-7 cells

To assess the iron chelation efficacy of the ligands in solution, the iron complexes of the weak iron chelator, calcein, were used. In this assay, the examined chelators compete with calcein for iron and the fluorescence of the free, dequenched calcein is proportional to their chelation efficacy in comparison to calcein. The iron chelation efficacy of the novel ligands was expressed as a percentage of the level of calcein de-quenching caused by the parent chelator, SIH (100%).

**Figure 3:**
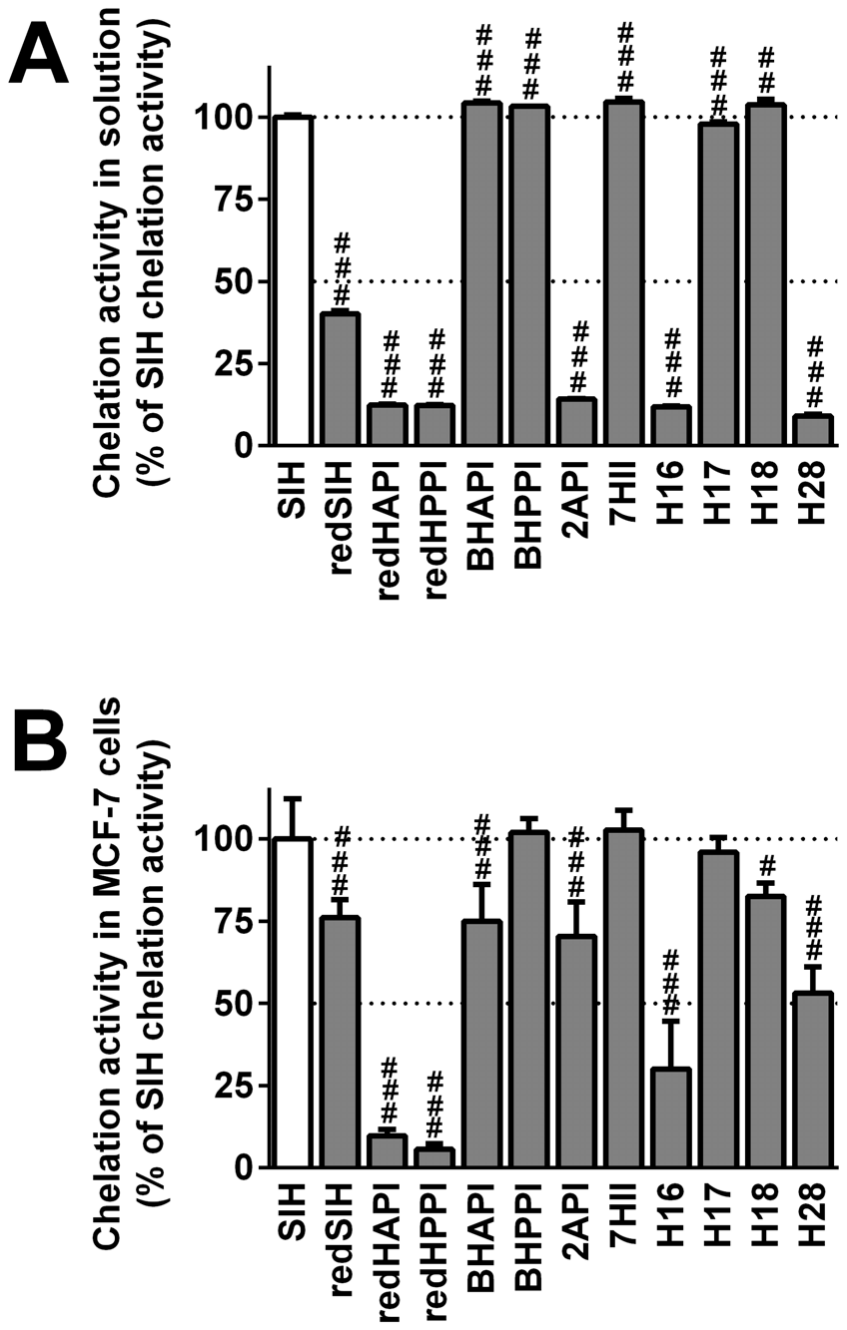
Iron chelation properties of the novel analogs in solution (A) and in MCF-7 cells (B). (**A**) The chelation dynamics of the new agents in solution were observed for 360 s using the calcein assay, and the agent was applied at *t* = 100 s. The fluorescence intensity of free calcein at *t* = 360 s was expressed as a percentage of that observed using the reference iron chelator, SIH. (**B**) The ability of the analogs to chelate “free” iron from the LIP in MCF-7 cells was measured using the calcein-AM assay. The fluorescence intensity of free calcein at *t* = 600 s was expressed as a percentage of that observed in the presence of SIH. Results are Mean ±SD (*n*≥3 experiments). Statistical significance (ANOVA): # *p*<0.05, ## *p*<0.01, ### *p*<0.001 compared to the reference chelator, SIH.

The reduction of the hydrazone bond in redSIH, redHAPI and redHPPI resulted in significantly (*p*<0.001) reduced iron chelating efficacies in solution (Fig. 3A). The brominated ligands, BHAPI and BHPPI, and the alkylated analogs, 7HII, H17 and H18 exhibited iron chelating activity similar to the reference agent, SIH (Fig. 3A). The 2-acetylpyridine derivative, 2API, was observed to have poor iron chelating efficacy in this assay relative to SIH. However, this may be due to the ability of the iron complex of 2API to oxidize calcein [54], as the iron complex of 2API was identified to act as a pro-oxidant (see below), and thus, resulted in decreased calcein fluorescence. Additionally, low chelation efficacy was also observed for the ligands, H16 and H28 (Fig. 3A), that possess an isopropyl or cyclohexyl group, respectively, adjacent to the hydrazone bond.

The efficacy of the ligands to permeate the cell membrane to gain access to the LIP was examined using the calcein-AM assay in iron-loaded MCF-7 cells (Fig. 3B). In these studies, the iron chelation efficacy of the synthesized ligands was expressed as a percentage of the efficiency of the parent chelator, SIH (100%). The ability of the chelators, BHPPI, 7HII and H17 to permeate the cell membrane and to bind iron from the calcein-AM detectable LIP did not significantly (*p*>0.05) differ from that of SIH (Fig. 3B). This was well correlated with their high chelation efficacy in solution (Fig. 3A). The ligands, redSIH, BHAPI, 2API, H18 and H28, exhibited moderate (50–80% relative to SIH), but significantly (*p*<0.05–0.001) decreased iron chelation efficacy in MCF-7 cells relative to SIH (Fig. 3B). In contrast, redHAPI, redHPPI and H16 displayed the poorest ability (<50% relative to SIH) to access and bind iron from the LIP (Fig. 3B) and this was in good correlation to their chelation activity in solution (Fig. 3A).

### 3. The effect of the chelators on the mobilization of cellular 59Fe and prevention of cellular 59Fe uptake from 59Fe2-Tf

**Figure 4:**
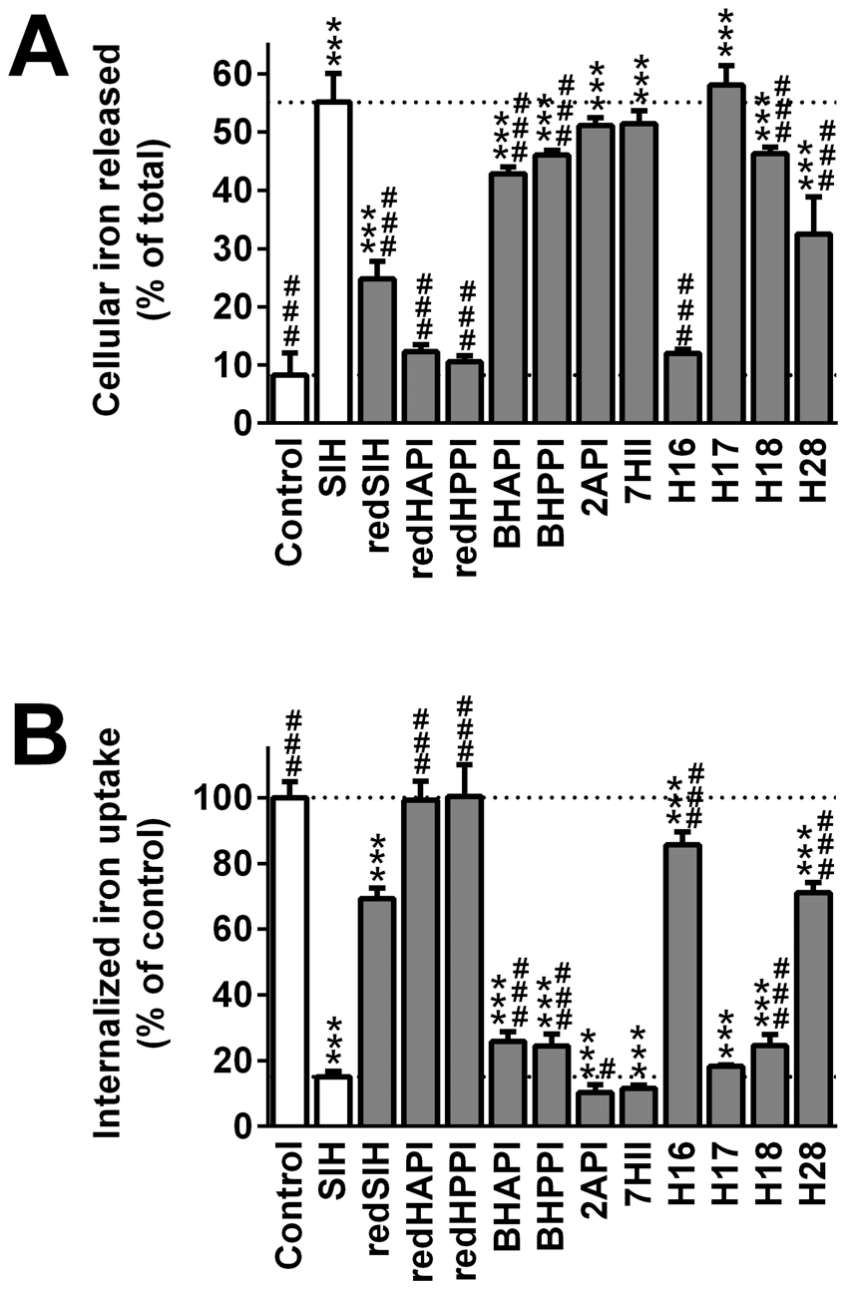
The effect of SIH and its analogs on ^59^Fe mobilization from pre-labeled MCF-7 cells (A) and on internalized ^59^Fe uptake from ^59^Fe_2_-transferrin (Tf) by MCF-7 cells (B). (**A**) The ability of the ligands to promote ^59^Fe mobilization from MCF-7 cells was performed by first prelabeling the cells with ^59^Fe_2_-Tf (0.75 µM) for 3 h/37°C, followed by washing and then reincubation for 3 h/37°C with either control medium alone, or control medium containing the chelator (25 µM). (**B**) Inhibition of ^59^Fe uptake from ^59^Fe_2_-Tf by MCF-7 cells by chelators was performed by incubating cells for 3 h/37°C with ^59^Fe_2_-Tf (0.75 µM) in the presence or absence of the chelator (25 µM). Results are Mean ±SD (*n*≥3 experiments). Statistical significance (ANOVA): * *p*<0.05, ** *p*<0.01, *** *p*<0.001 compared to the control (untreated) group, and # *p*<0.05, ## *p*<0.01, ### *p*<0.001 compared to the reference chelator, SIH.

To examine the ability of the novel ligands to mobilize intracellular ^59^Fe from MCF-7 cells, ^59^Fe efflux experiments were performed using established techniques [34], [45]. The novel ligands were compared to control medium containing no added chelator and also to the parent analog, SIH (Fig. 4A). The control medium showed limited ability to mobilize cellular ^59^Fe, resulting in the release of 8% of cellular ^59^Fe (Fig. 4A). In contrast, SIH displayed high ^59^Fe mobilization efficacy, mediating the release of 55% of cellular ^59^Fe (Fig. 4A). The ligands, BHAPI, BHPPI, 2API, 7HII, H17 and H18 were highly effective in mediating ^59^Fe mobilization and resulted in the release of 43–58% of cellular ^59^Fe (Fig. 4A). The agents, redSIH and H28 demonstrated significantly (*p*<0.001) increased ^59^Fe mobilization compared to the control. However, their ^59^Fe mobilization efficacy was approximately half that of SIH (Fig. 4A). The ^59^Fe mobilization efficacy of redHAPI, redHPPI and H16 were poor and comparable to the untreated control (Fig. 4A). In general, the results of this assay correlated well with the observed iron-chelation efficacies of these analogs in solution (Fig. 3A) and in the cell-based calcein-AM assay (Fig. 3B). The only notable exception was 2API, which demonstrated high activity at mobilizing cellular ^59^Fe (Fig. 4A), which was in contrast to the iron chelation assay in solution (Fig. 3A). As noted previously, this could be due to its pro-oxidative effects on calcein [54].

As the iron chelation efficacy and cytotoxic activity of a ligand are due to both its ability to mobilize cellular Fe, but also, inhibit Fe uptake from Tf [34], the ability of the chelators to prevent the cellular uptake of ^59^Fe from ^59^Fe_2_-Tf was determined and expressed as a percentage of the untreated control (Fig. 4B). As observed in the ^59^Fe mobilization experiments, the parent chelator, SIH, demonstrated high ^59^Fe chelation efficacy and inhibited ^59^Fe uptake to 15% of the control (Fig. 4B).

Importantly, those ligands that showed high ^59^Fe mobilization efficacy (Fig. 4A) were also highly effective at inhibiting the uptake of ^59^Fe from ^59^Fe_2_-Tf (Fig. 4B). For example, the ligands, BHAPI, BHPPI, 2API, 7HII, H17 and H18, that demonstrated high ^59^Fe mobilization activity, were able to limit ^59^Fe uptake to 10–26% of the control (Fig. 4B). In contrast, the compounds, redSIH, redHAPI, redHPPI, H16, and H28, showed limited ability to prevent ^59^Fe uptake, inhibiting it to>70% of the control (Fig. 4B).

### 4. Examination of the ability of the iron-chelator complexes to catalyze the oxidation of ascorbate

It has been previously observed that the cytotoxic effects of some iron chelators is due not only to their ability to bind cellular iron, but also to form redox-active iron complexes [12], [46], [55]. Thus, we examined whether the iron complexes of our novel ligands were able to redox cycle by assessing their ability to mediate the oxidation of ascorbate by standard methods [46], [48]. The ability of the iron complexes to catalyze the oxidation of ascorbate was expressed as a percentage of the control (ascorbate with “free” Fe^3+^).

**Figure 5:**
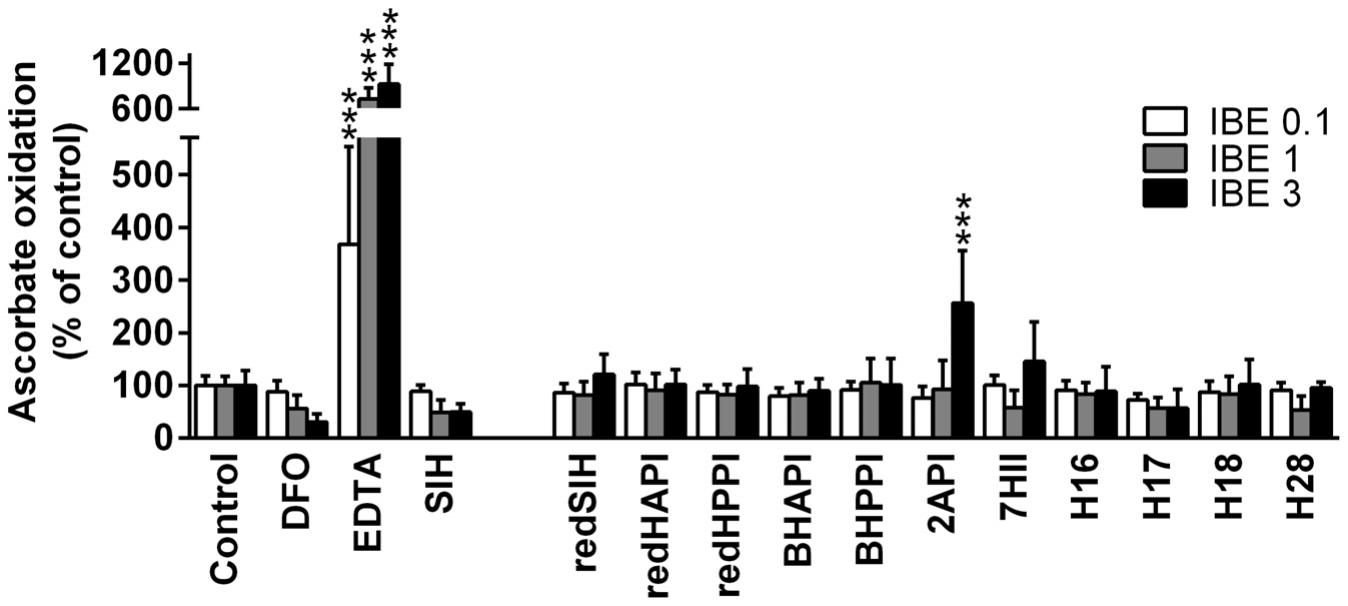
Effects of SIH and its analogs on iron-induced oxidation of ascorbic acid in a buffered solution (pH 7.4). Chelators were assayed at iron binding equivalents (IBE) of 0.1 (excess of Fe), 1 (iron-chelator complexes with a fully filled coordination sphere) and 3 (excess of free chelator). DFO and EDTA were used as negative and positive control chelators, respectively. The results are expressed as a percentage of the control group in the absence of chelator (100%). Results are Mean ±SD (*n*≥3 experiments). Statistical significance (ANOVA): * *p*<0.05, ** *p*<0.01, *** *p*<0.001 as compared to the control group (iron with ascorbate).

The chelators, DFO and EDTA, were used as negative (anti-oxidative) and positive (pro-oxidative) controls, respectively [46], [49]. As previously observed, the Fe complex of DFO demonstrated a typical anti-oxidative profile [56], resulting in decreased levels of ascorbate oxidation at an IBE of 3 (excess DFO) than at an IBE of 0.1 (excess iron; Fig. 5). In contrast, the iron complex of EDTA exhibited a pro-oxidative effect and mediated higher levels of ascorbate oxidation at an IBE of 3 relative to that at 0.1 (Fig. 5). In fact, at an IBE of 3, the iron complex of EDTA increased the oxidation of ascorbate to 924% of the control.

The iron complex of the parent chelator, SIH, exhibited anti-oxidant activity similar to that of the iron complex of DFO (Fig. 5). All of the iron complexes of the novel ligands, with the exception of 2API, demonstrated neither anti-oxidant nor pro-oxidative effects and were comparable to the control. The iron complex of the pyridine derivative, 2API, was the only Fe complex that showed pro-oxidative effects and significantly (*p*<0.001) increased ascorbate oxidation to 256% relative to the control at an IBE of 3 (Fig. 5).

### 5. Prevention of oxidative injury induced by hydrogen peroxide

**Figure 6:**
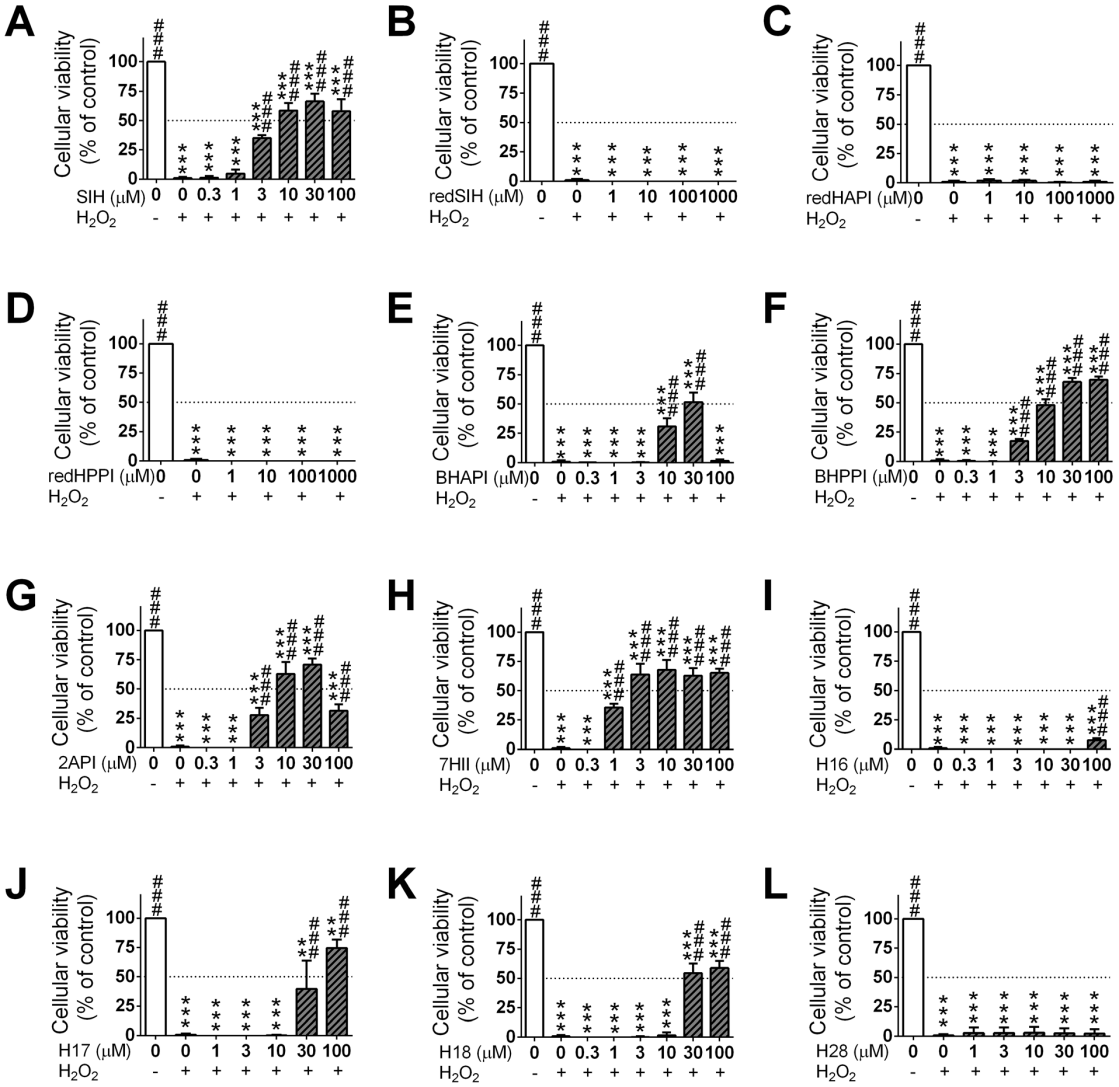
Protective effects of the chelator, SIH (A), and the new analogues (B–L). The ability of the SIH derivatives to protect H9c2 cardiomyoblast cells against oxidative injury were evaluated using a 24 h/37°C incubation of the cells with H_2_O_2_ (200 µM) and the novel analogs (0.3–1000 µM). Results are Mean ±SD (*n*≥4 experiments). Statistical significance (ANOVA): * *p*<0.05, ** *p*<0.01, *** *p*<0.001 compared to the control (untreated) group, and # *p*<0.05, ## *p*<0.01, ### *p*<0.001 compared to the H_2_O_2_ group.

The ability of the ligands to act as protective agents in a model of oxidative stress was then examined by assessing the cellular viability of H9c2 cardiomyoblast cells upon a 24 h co-incubation of the chelators with H_2_O_2_ (200 µM). These results are shown in Fig. 6 and summarized in Table 3. In these experiments, the EC_50_ value is calculated which represents the concentration that reduced the cytotoxicity induced by hydrogen peroxide (200 µM) to 50% of the untreated control after a 24 h/37°C incubation with H9c2 cells. SIH was used as a positive control and resulted in an EC_50_ value of 7.63±1.38 µM (Table 3).

**Table 3:** Protective and cytotoxic effects of the synthesized SIH derivatives and their calculated “selectivity ratios”.

Chelator	EC_50_ H9c2 (µM)	IC_50_ H9c2 (µM)	IC_50_ MCF-7 (µM)	Selectivity Ratio
**SIH**	7.63±1.38	49.47±1.77	4.21±1.05	11.75
**redSIH**	N/A	39.59±5.11	279.97±53.17	0.14
**redHAPI**	N/A	83.96±2.76	133.47±28.76	0.63
**redHPPI**	N/A	226.12±6.31	197.86±13.09	1.14
**BHAPI**	30.34±7.23	6.99±0.82	1.06±0.46	6.59
**BHPPI**	17.18±4.39	6.31±0.59	0.83±0.50	7.60
**2API**	8.48±3.11	3.07±0.55	2.92±0.67	1.05
**7HII**	2.68±1.30	0.62±0.17	0.38±0.11	1.63
**H16**	N/A	>100	153.67±24.20	-
**H17**	42.57±7.94	32.60±1.09	2.27±0.14	14.36
**H18**	27.76±3.90	7.40±2.13	0.49±0.18	15.10
**H28**	N/A	85.37±12.90	42.41±3.15	2.01

The EC_50_ values (concentration that reduced the cytotoxicity induced by H_2_O_2_ (200 µM) to 50% of the untreated control) were calculated after a 24 h incubation with non-tumorigenic H9c2 cardiomyoblasts. The IC_50_ values (concentration that reduced the cellular viability or proliferation to 50% of the untreated control) were calculated after a 72 h incubation with H9c2 cardiomyoblasts or MCF-7 breast cancer cells. Selectivity ratios were calculated *via* IC_50_ H9c2 cells/IC_50_ MCF-7 cells. Mean ± SD; *n*≥4 experiments. N/A - the EC_50_ value was not achieved within the studied concentration range (no protection).

Of all the novel ligands synthesized, the analog that displayed the highest level of cytoprotective activity was 7HII, with an EC_50_ value of 2.68±1.30 µM (Table 3). In fact, 7HII demonstrated significantly (*p*<0.001) greater protection against hydrogen peroxide-induced cytotoxicity than the parent chelator, SIH. Although the iron chelators, BHAPI, BHPPI, 2API, H17 and H18 also prevented peroxide-induced cytotoxicity (EC_50_: 8.48–42.57 µM), their EC_50_ values were higher than that of SIH. The ligands, redSIH, redHAPI, redHPPI, H16 and H28 did not display protective activity against peroxide-induced cytotoxicity in the concentration range examined.

### 6. Cytotoxicity studies in H9c2 cardiomyoblast cells

**Figure 7:**
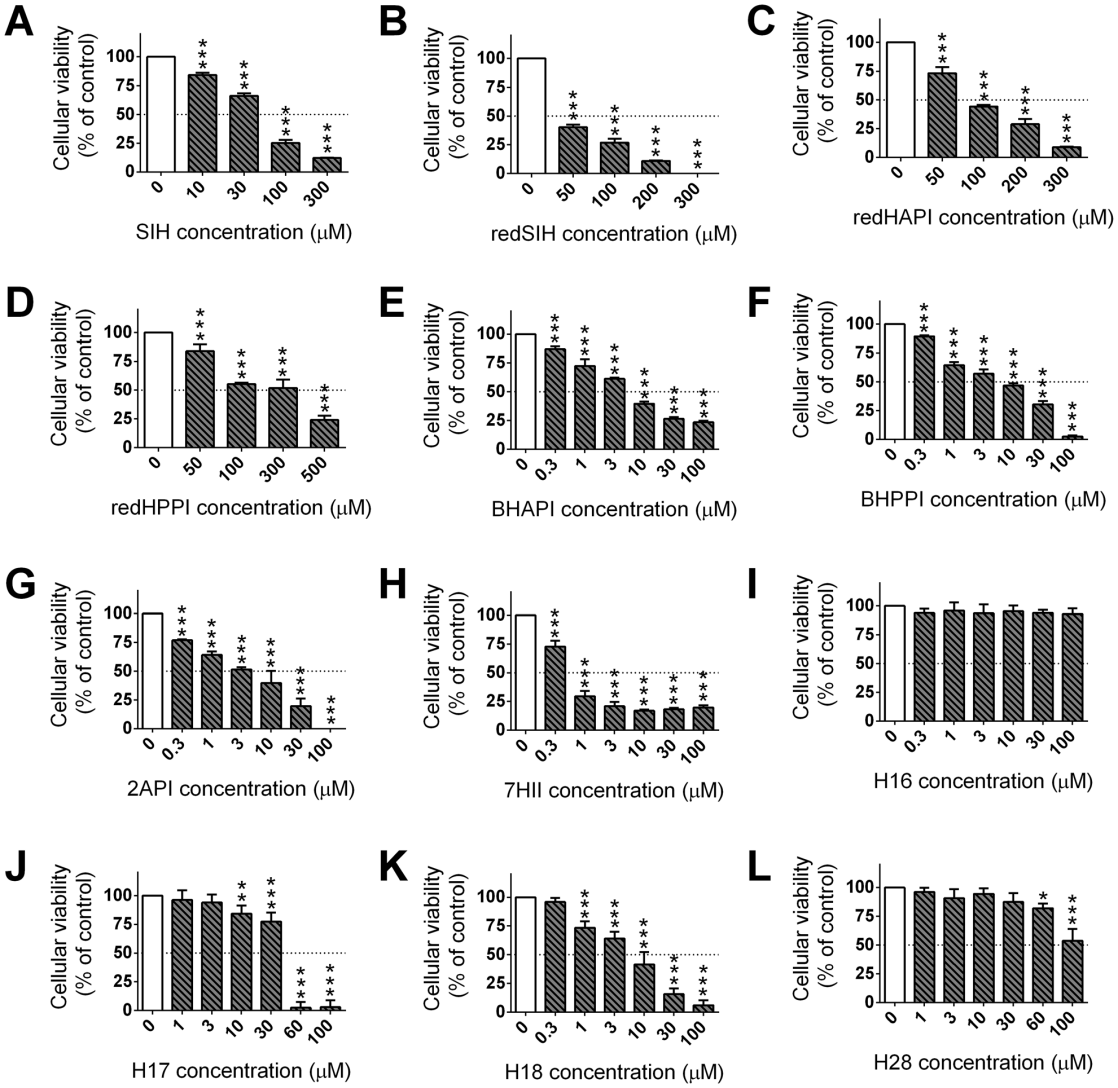
Cytotoxic effects of the chelator, SIH (A), and the new analogues (B–L), using non-tumorigenic H9c2 cardiomyoblasts. The effect of the analogs (0.3–300 µM) on the cellular viability of H9c2 cardiomyoblasts were performed using a 72 h/37°C incubation. Results are Mean ±SD (*n*≥4 experiments). Statistical significance (ANOVA): * *p*<0.05, ** *p*<0.01, *** *p*<0.001 as compared to the control (untreated) group.

The selectivity of the novel ligands was then examined after a 72 h incubation with the non-tumorigenic H9c2 cardiomyoblast cell line (Fig. 7; Table 3). The parent chelator, SIH, was examined as a control and demonstrated an IC_50_ value of 49.47±1.77 µM (Table 3).

Of the synthesized analogs, redHAPI, redHPPI, H16 and H28 were the least toxic agents, with IC_50_ values>80 µM. The ligands, redSIH and H17, showed comparable cytotoxicity to H9c2 cardiomyoblasts as the parent chelator, SIH. The other studied ligands, BHAPI, BHPPI, 2API, 7HII, H18, were more toxic than the chelator, SIH, with IC_50_ values ranging from 0.62 µM to 7.40 µM. The most cytotoxic agent was 7HII with an IC_50_ value of 0.62±0.17 µM (Table 3; Fig. 7).

### 7. Cytotoxic effects of SIH derivatives on MCF-7 cells

**Figure 8:**
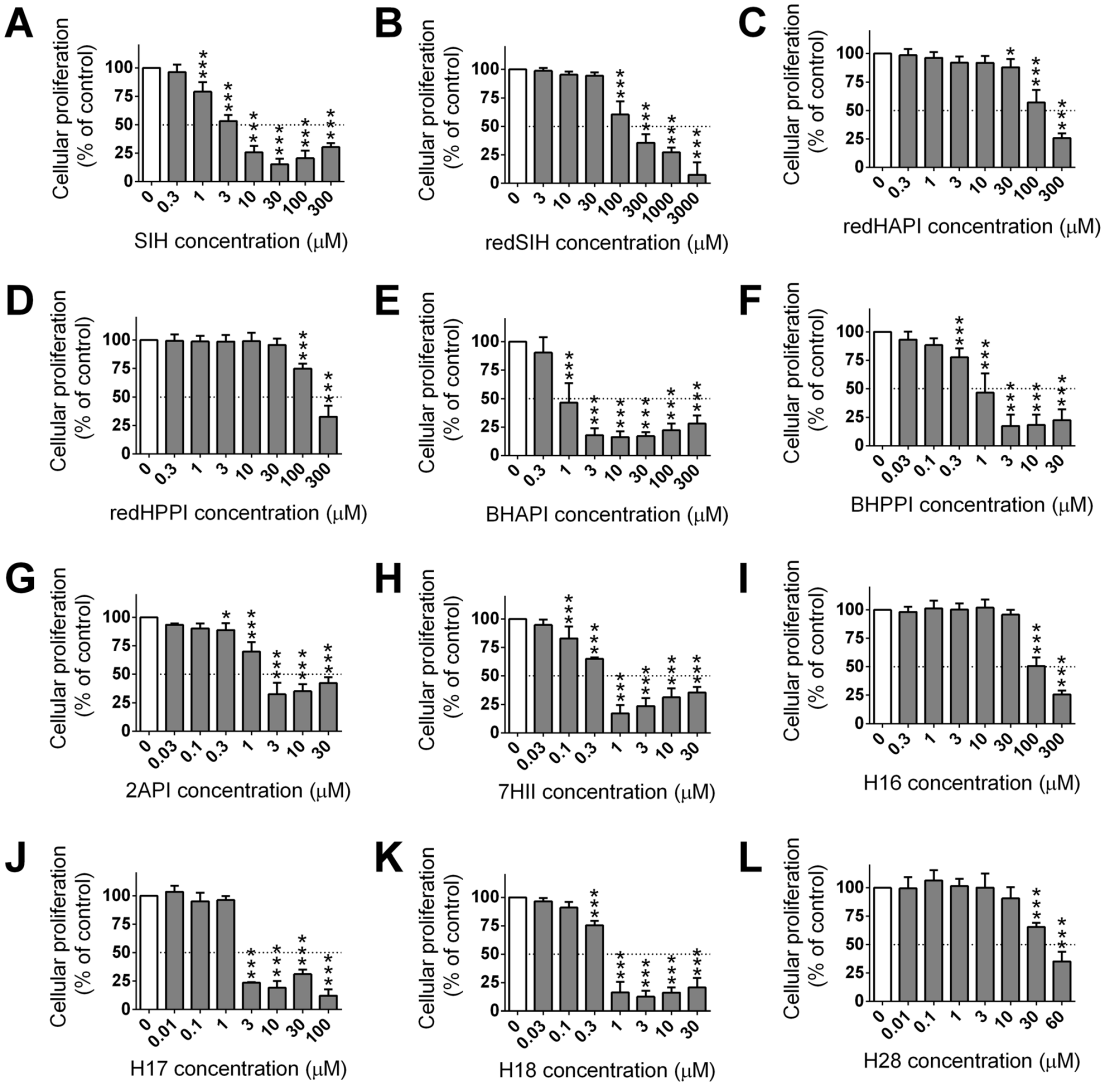
Cytotoxic effects of the chelator, SIH (A), and the new analogues (B–L) against MCF-7 breast cancer cells. For the determination of their cytotoxic activity, MCF-7 breast adenocarcinoma cells were incubated with the analogs (0.01–3000 µM) for 72 h/37°C. Results are Mean ±SD (*n*≥4 experiments). Statistical significance (ANOVA): * *p*<0.05, ** *p*<0.01, *** *p*<0.001 as compared to the control (untreated) group.

The cytotoxic effects of the SIH derivatives were studied in MCF-7 breast adenocarcinoma cells following a 72 h incubation. The parent chelator, SIH, was used as a control and demonstrated moderate cytotoxic activity (IC_50_: 4.21±1.05 µM; Table 3; Fig. 8), similar to that previously observed [31].

The analogs containing a reduced hydrazone bond (redSIH, redHAPI and redHPPI) or an isopropyl group adjacent to this bond (H16) exhibited poor cytotoxic activity (IC_50_>100 µM). The chelator, H28, with a bulky cyclohexyl group in close proximity to the hydrazone bond demonstrated intermediate cytotoxic effects, with an IC_50_ of 42.41±3.15 µM. The remaining agents, BHAPI, BHPPI, 2API, 7HII, H17 and H18, showed increased cytotoxic activity (IC_50_: 0.38–2.92 µM; Table 3) relative to SIH (Table 3). The greatest level of cytotoxic activity was observed with the indanone derivative, 7HII (IC_50_ = 0.38±0.11 µM).

To provide insight into the selectivity of the cytotoxic effects of the novel ligands, which is crucial for potential anti-cancer agents, their IC_50_ values in H9c2 cells and their IC_50_ values in MCF-7 cells were compared by calculating a “selectivity ratio”, namely IC_50_ H9c2/IC_50_ MCF-7 cells (Table 3). SIH had a selectivity ratio of 11.75. The analogs, redSIH and redHAPI, with reduced hydrazone bonds had lower IC_50_ values in H9c2 cardiomyoblasts than in MCF-7 cancer cells, indicating greater cytotoxic activity in the former. Relative to SIH, this resulted in a marked decrease in the selectivity ratio to 0.14 and 0.63, respectively (Table 3). The ligands, redHPPI, 2API, 7HII and H28, showed somewhat similar cytotoxic activity in both the MCF-7 and H9c2 cell-types leading to selectivity ratios that were far less than SIH, and which ranged between 1.05 and 2.01. On the other hand, the bromine-substituted chelators (BHAPI and BHPPI) demonstrated selective activity against MCF-7 breast cancer cells relative to the H9c2 cell-type, although their selectivity ratios were approximately half that observed for SIH, *viz.*, 6.59 and 7.60, respectively (Table 3). The analogs that demonstrated the greatest selectivity profile against MCF-7 cells relative to H9c2 cells were the propyl (H17) and isobutyl (H18) derivatives of SIH, which were more active than SIH itself, demonstrating selectivity ratios of 14.36 and 15.10, respectively (Table 3).

## Discussion

Aroylhydrazones represent an intriguing group of chelators that exhibit a variety of biological effects associated with their ability to influence cellular iron levels [27], [33], [57]. The aim of the present study was to synthesize and evaluate the biological activity of a series of new analogs of the well-established iron-binding ligand, SIH, with respect to their: (***1***) stability in plasma, (***2***) cytotoxic effects; (***3***) ability to protect cells against oxidative injury; and (***4***) cytotoxicity to H9c2 non-tumorigenic cardiomyoblast cells. The iron chelation activity, ability to mobilize cellular ^59^Fe, efficacy to inhibit ^59^Fe uptake from ^59^Fe_2_-Tf, and the redox activity of the iron complexes of the novel analogs were also determined, as these properties are crucial factors involved in their biological activity [34], [35]. The primary goal was to further characterize the structure-activity relationships of SIH-related aroylhydrazones for the future rational design of compounds with therapeutic potential.

### 1. Reduction of the hydrazone bond

First, we probed the role of the hydrazone bond itself, as it is prone to hydrolysis and is a site of instability in this class of compounds [58]. Previous studies suggested that structurally- related compounds with a reduced C = N bond retained their chelation properties [59]. In fact, these reduced compounds inhibited the iron-induced generation of hydroxyl radicals and protected murine dermal fibroblasts against UV-induced lipid peroxidation and UV-induced cytotoxicity [59]. Thus, we examined the effect of the reduction of the hydrazone bond of the chelators, SIH, HAPI and HPPI, as these ligands previously exhibited cardioprotective [33] and cytotoxic [31] activity.

The results of the present study revealed that the reduced analogs were relatively non-toxic against both tumorigenic MCF-7 cells and non-tumorigenic H9c2 cardiomyoblasts (Table 3). The cytotoxicity of redHAPI and redHPPI were approximately one order of magnitude lower than those of the parent chelators (HAPI and HPPI, respectively) [31], [33], while the cytotoxic activity of redSIH towards H9c2 cells was similar to that of SIH (Table 3). This effect could be caused by the increased stability of redSIH (Fig. 2B) compared to SIH (Fig 2A), and therefore, the prolonged exposure of cells to intact redSIH compensated for the reduced (yet significant) iron chelation activity. Reduction of the hydrogen bond in redSIH, redHAPI and redHPPI led to a marked decrease in their selectivity ratios (0.14–1.14) relative to SIH (11.75; Table 3). In fact, these agents containing a reduced hydrazone bond had the lowest selectivity ratios of all analogues examined in this investigation. Furthermore, these latter compounds lost the ability to protect H9c2 cells against oxidative stress relative to SIH (Table 3) [33]. This lack of protection against oxidative stress is likely due to their limited iron chelation (Fig. 3) and ^59^Fe mobilization efficacy (Fig. 4A). Of the reduced analogs, only redSIH retained limited chelation activity (Figs. 3 and 4). Therefore, the presence of the hydrazone bond is an important criterion for the cardioprotective and cytotoxic effects of these aroylhydrazones. The loss of iron chelation efficacy of the reduced analogs may be a result of the altered molecular spatial arrangement of the ligating groups due to the free rotation of the single C-N bond, or the decreased electron density on the chelating nitrogen due to its transition from sp^2^ to sp^3^ orbital hybridization.

### 2. Bromination of the phenyl ring

The introduction of a halogen into the structure of a molecule enhances its lipophilicity (Table 2), which can potentially facilitate its permeation into cells. The halogen substitution, due to its inductive electron-withdrawing effects, may also influence the stability of the hydrazone bond and the ability of the compound to chelate metal ions. Indeed, a previously synthesized chlorinated HAPI derivative (*i.e.*, (*E*)-*N′*-[1-(5-chloro-2-hydroxyphenyl)ethylidene]isonicotinoylhydrazide; CHAPI), showed greater hydrolytic stability than HAPI and moderate cytotoxic activity (IC_50_ = 0.65±0.07 µM against MCF-7 cells) [31]. Therefore, the brominated analog, BHAPI, bearing a bromine instead of chlorine, and its homolog, BHPPI (Fig. 1), was prepared to evaluate the influence of halogenation on the cardioprotective and cytotoxic activity of these chelators. The stability of BHAPI and BHPPI was similar to the chloro derivative, CHAPI. However, the presence of bromine instead of the chlorine substituent increased the chelating efficiency of these compounds in cells from approximately 50% for CHAPI, to 75% and 100% for BHAPI and BHPPI, respectively. Both BHAPI and BHPPI showed comparable iron chelation efficacy to SIH in solution, as well as in cells (Fig. 3). The cytotoxic activity of these brominated analogs against MCF-7 cells was greater than that found for SIH (Table 3). Further, both BHAPI and BHPPI showed greater cytotoxic activity against MCF-7 breast cancer cells relative to non-tumorigenic, H9c2 cardiomyoblasts, although their selectivity ratios were approximately half that observed for SIH (Table 3). In addition, BHAPI and BHPPI were less effective than SIH when assessing the ability of these agents to prevent the cytotoxicity induced by H_2_O_2_ in H9c2 cardiomyoblasts (Table 3). Similar results were previously observed for the chlorine derivative, CHAPI [33].

### 3. Exchange of phenol for pyridine

The ligand, 2API, which contains a pyridine nitrogen as a donor atom instead of the phenolic oxygen, was prepared to examine the effect of alterations of the donor atom set from *O*, *N*, *O* to *N*, *N*, *O* on their biological activity. The main reason for this structural modification was that exchanging a hard base ligand (phenolic oxygen) for a softer base (nitrogen) could markedly alter the ability of such a compound to bind Fe^3+^/Fe^2+^. In addition, structurally similar hydrazones derived from pyridine-2-carbaldehyde gained attention in the treatment of iron overload diseases [37], [38]. The cytotoxic activity of 2API was similar in MCF-7 breast cancer cells and non-tumorigenic H9c2 cells, with the selectivity ratio decreasing markedly (to 1.05) relative to that observed with SIH (11.75; Table 3). This observation may be explained by the redox activity of 2API, as it was the only analog that exhibited significant pro-oxidative activity in the ascorbate oxidation assay (Fig. 5). In fact, previous studies reported the reversible Fe^2+/3+^ redox couple of the iron complex of 2API [60] and the current investigation demonstrates its ability to oxidize ascorbate.

The iron chelation efficacy and ^59^Fe mobilization activity of 2API in cells was marked, with the ligand being generally comparable to SIH (Figs. 3B, 4A, 4B). In contrast, the iron chelation activity of 2API in solution did not correlate with the results of cellular experiments (Fig. 3A), which may be explained by the pro-oxidative effects of 2API. It is possible the ability of the 2API iron complex to redox cycle may have interfered in the solution-based calcein assay, as it is known that the fluorescence of free calcein decreases in an oxidative environment [54]. Whereas the unaltered sensitivity of the calcein-AM assay in cells (Fig. 3B) with regards to 2API, may be due to the redox buffering capacity provided by glutathione and other intracellular anti-oxidative systems [61] that maintain calcein sensitivity. In summary, the alteration of the donor atom set from *O*, *N*, *O* to *N*, *N*, *O* in 2API resulted in the formation of a redox active iron complex with decreased selectivity against MCF-7 breast cancer cells. However, the exact mechanism of action of this compound remains to be elucidated.

### 4. Branching, prolongation or cyclization of the alkyl chain adjacent to the hydrazone bond

In a previous investigation, we found that the presence of an alkyl chain adjacent to the hydrazone bond did not significantly increase the cytotoxic activity of the ketone-derived hydrazones, HAPI and HPPI, compared to SIH [31]. In the current study, we synthesized the analogs, 7HII, H16, H17, H18 and H28, to evaluate the influence of alkyl chain length and branching on biological activity.

The 7-hydroxyindanone derivative, 7HII, contains an extra five-membered ring relative to SIH and showed comparable iron chelating and ^59^Fe mobilization efficacy (Figs. 3, 4). The cyclization of the alkyl chain, and hence, its increased rigidity, improved its hydrolytic stability (Fig 2H) and also its ability to protect cells against oxidative stress compared to SIH, with 7HII being the most effective ligand screened in this regard (Table 3). However, this structural change in 7HII resulted in significantly higher cytotoxicity towards H9c2 cells and a marked drop in the selectivity ratio relative to SIH (Table 3). Therefore, this structural modification resulted in unfavorable biological activity.

The ligand, H16, bears an additional isopropyl chain at the α-position from the hydrazone bond relative to SIH (Fig. 1). This modification was intended to: (***1***) protect the hydrazone bond against hydrolysis [62]; and (***2***) increase lipophilicity, which is known to enhance cellular permeability of aroylhydrazone ligands [34]. However, this structural modification in H16 resulted in similar stability in plasma as SIH and a marked loss of its iron chelation activity relative to SIH. This effect may be due to steric hindrance around the hydrazone bond mediated by the bulky branched isopropyl group that potentially reduces binding to iron. Notably, consistent with the loss of iron-binding, the cytotoxic activity of H16 was very low in H9c2 and MCF-7 cells and did not show any protective effects against H_2_O_2_ (Table 3).

To examine whether the effect of the isopropyl chain of H16 was caused by steric hindrance close to the hydrazone bond, compound H17, with an unbranched propyl chain, was prepared. Interestingly, this ligand was even more stable in plasma (Fig. 2J) than its homolog HPPI [33]. Furthermore, the iron chelation and ^59^Fe mobilization efficacy of H17 was similar to SIH, with the compound showing selective cytotoxic activity against MCF-7 cancer cells relative to non-tumorigenic H9c2 cardiomyoblasts [31], [33]. In fact, the selectivity ratio of H17 (14.36) was greater than that found for SIH (11.75), demonstrating its potential. We were also interested to examine whether H18, with an isobutyl substituent adjacent to the hydrazone bond (Fig. 1), would retain the favorable activity of H17. In contrast to H16, H18 is branched at the β-position in relation to the imine carbon and led to the ligand maintaining hydrolytic stability, iron chelation efficacy in solution and also in cells relative to SIH (Figs. 2J, 3, 4). This structural change increased the cytotoxic activity of H18 against both MCF-7 tumor cells and H9c2 cardiomyoblasts relative to SIH and H17 (Table 3). However, notably, H18 had the best selectivity ratio of all the studied compounds (*i.e.*, 15.10).

To further examine the structure-activity relationships of bulky substituents close to the hydrazone bond, compound H28, with a cyclohexyl group, was prepared. As in the case of H16, this modification did not improve the low hydrolytic stability observed with SIH (Fig. 2L). Also, the iron chelation efficacy of H28 was markedly decreased (Figs. 3, 4). Furthermore, in comparison with H16, the cytotoxic activity of H28 was greater in both MCF-7 and H9c2 cells, leading to an unfavorable selectivity ratio of 2.01 which was much less than SIH. In addition, the cardioprotective activity of H28 against H_2_O_2_ was completely abolished, which is consistent with the low iron chelation efficacy of H28.

Thus, the alkyl chain on the imine carbon markedly influenced the activity of such hydrazones. Prolonged linear or iso-branched alkyl groups increased their anti-cancer potential, while branching or cyclization in close proximity to the hydrazone bond dramatically decreased their chelation ability and, consequently, decreased their cytotoxic activity against MCF-7 cells and their ability to protect H9c2 cells against oxidative injury.

### 5. Conclusions

In this study, we identified several structural parameters important for the design of aroylhydrazone iron chelators. First, the hydrazone bond is essential for chelation activity. Second, bromination of the phenyl ring does not have any beneficial effect due to increased non-selective cytotoxic activity against non-tumorigenic H9c2 cardiomyoblasts. Third, exchange of the chelating phenolic hydroxyl (a hard base) for a pyridine nitrogen (softer base) resulted in increased non-selective cytotoxic activity, the mechanism of which is not exactly known. Finally, and most significantly, the exchange of the aldimine hydrogen in SIH for a longer unbranched or iso-branched alkyl group is a favorable modification to increase the stability and anti-cancer potential of such hydrazones. The most promising compounds identified in this study are the propyl-containing analog, H17, and isobutyl-containing derivative, H18, which possessed the highest selectivity ratios. These compounds warrant further investigation.

## Supporting Information

Data S1 **Raw data underlying the findings in this study.**
(ZIP)
